# Comparative transcriptome analysis of molecular mechanisms underlying adventitious root developments in Huangshan Bitter tea (*Camellia gymnogyna Chang*) under red light quality

**DOI:** 10.3389/fpls.2023.1154169

**Published:** 2023-03-21

**Authors:** Hao Fu, Xu Wei, Qian Chen, Shunyuan Yong, Qinjin Liu, Jiangbo Dang, Di Wu, Guolu Liang, Qigao Guo

**Affiliations:** ^1^ Key Laboratory of Horticulture Science for Southern Mountains Regions of Ministry of Education, College of Horticulture and Landscape Architecture, Southwest University, Chongqing, China; ^2^ Academy of Agricultural Sciences of Southwest University, State Cultivation Base of Crop Stress Biology for Southern Mountainous Land, Chongqing, China; ^3^ Citrus Research and Education Center, University of Florida, Lake Alfred, FL, United States; ^4^ Chongqing Institute of Ancient Tea Plant and Product, Chongqing, China

**Keywords:** Camellia gymnogyna Chang, adventitious root formation, transcriptome analysis, light quality, plant phytohormone signal transduction

## Abstract

As the formation of adventitious roots (AR) is an important component of in vitro regeneration of tea plants, the propagation and preservation of Huangshan Bitter tea (*Camellia gymnogyna Chang*) cuttings have been hindered due to its lower rooting rate. As light is a crucial environmental factor that affects AR formation, this study aimed to investigate the special role of red light (RL) in the formation of AR in Huangshan Bitter tea plants, which has not been well understood. Huangshan Bitter tea plants were induced with white light (control, WL) and red light (660 nm, RL) qualities 36 days after induced treatment (DAI) to investigate dynamic AR formation and development, anatomical observation, hormones content change, and weighted gene co-expression network analysis (WGCNA) of the transcriptome. Results showed that RL promoted the rooting rate and root characteristics compared to WL. Anatomical observations demonstrated that root primordium was induced earlier by RL at the 4 DAI. RL positively affected IAA, ZT and GA_3_ content and negatively influenced ABA from the 4 to 16 DAI. RNA-seq and analysis of differential expression genes (DEGs) exhibited extensive variation in gene expression profiles between RL and WL. Meanwhile, the results of WGCNA and correlation analysis identified three highly correlated modules and hub genes mainly participated in 'response to hormone', 'cellular glucan metabolic progress', and 'response to auxin'. Furthermore, the proportion of transcription factors (TFs) such as ethylene response factor (*ERF*), myeloblastosis (*MYB*), basic helix-loop-helix (*bHLH*), and WRKYGQK (*WRKY*) were the top four in DEGs. These results suggested that the AR-promoting potential of red light was due to complex hormone interactions in tea plants by regulating the expression of related genes. This study provided an important reference to shorten breeding cycles and accelerate superiority in tea plant propagation and preservation.

## 1 Introduction

Tea is the most widely consumed non-alcoholic beverage in the world (Chen et al., 2021), which offers a plethora of health benefits such as anti-oxidant, anti-cancer, anti-cardiovascular disease, anti-allergic activities and even memory improvement (Brickman et al., 2014; Yu et al., 2020). Huangshan Bitter tea (*Camellia gymnogyna Chang*) is a precious ancient tea plant discovered in 2021 at Yibin City, Sichuan Province, China. It is a rare germplasm resource for studying the distribution of tea group plants and the relationship between species and climatic conditions. Tissue culture provides new support that can accelerate breeding, save breeding space, and preserve rare germplasm resources, which is vital for developing new varieties of Huangshan Bitter tea and conducting quality research.

The formation of adventitious roots (AR) is crucial for successful tea seedling propagation, as the agricultural practice often result in production losses due to slow or non-rooted cuttings. Regulating light qualities has been shown to be an effective strategy that significantly contributes to AR formation (Pashkovskiy et al., 2021). In plants, many photoreceptors receive light signals and transmit them to various biological processes to guide root development (Kumari and Panigrahi, 2019; Li et al., 2017). Red light has recently gained attention in many fields of study. Studies have shown that red light can induce more lateral roots and enhance the number and length of roots in *S.kakudensis* and *Picea abies* (Alallaq et al., 2020; Manivannan et al., 2021). However, limited reports exist on the effects of red light on the AR process of tea plants, hindering improvements to propagation. A comprehensive understanding of the mechanisms underlying the AR process is essential for the utilization and conservation of Huangshan Bitter tea.

In recent years, the importance of hormones in the formation of AR has been revealed, as they influence cell fate and cell specialization (Woodward and Bartel, 2005; Maes et al., 2008). Auxin plays a crucial role in the transformation of parenchymal cells into metabolically active embryonic cells through dedifferentiation, leading to the formation of a potential root primordial point (Garrido et al., 2002; de Dorlodot et al., 2007). Cytokinin (CK) further regulates embryonic cells to form a meristem cell group *via* cell division, which becomes a visible root primordium (Gonzalez-Rizzo et al., 2006). The root primordium continues to grow into an AR through cell differentiation (Lucas et al., 2013). Interestingly, the effects of gibberellic acid (GA), abscisic acid (ABA) and jasmonic acid (JA) on AR formation depend on the plant species. For instance, GA promotes AR formation in pea (*Pisum sativum*) but inhibits it in *hybrid aspen* and *Arabidopsis* Gutierrez et al., 2012; Rasmussen et al., 2015; Zeng et al., 2021; Li et al., 2022). Therefore, the role of hormones in AR formation needs to be further explored through classical molecular biology techniques combined with high-throughput sequencing technology, to provide new insights into the functions of hormones in AR formation and development.

In this study, in order to solve the low rooting rate of tissue culture seedlings of Huangshan Bitter tea, we designed a laboratory experiment that investigated the dynamic formation and development processes of AR under red (660 nm) and white (control) light qualities. We utilized transcriptome sequencing and weighted gene co-expression network analysis (WGCNA) to identify co-expressed gene modules and hub genes, and measured the dynamic change of phytohormones and their association with genes in the hormonal pathway. Furthermore, several important TFs associated with AR development were identified. Overall, our findings provide valuable insights into improving the breeding of high-quality tea plants varieties.

## 2 Materials and methods

### 2.1 Plant materials, cultivated condition, and treatments

This study was conducted in the artificial climate chamber of the Key Laboratory of Horticulture Science for Southern Mountains Regions of Ministry of Education, College of Horticulture and Landscape Architecture, Southwest University, Chongqing, China in September 2019. The plant material used in the experiment was 3 -5 cm tissue culture seedlings of Huangshan Bitter tea (*Camellia gymnogyna Chang*), which were sowed in a tissue culture bottle filled with the substrate (1/2 MS + 1.0 mg·L^−1^ IBA + 2.0 mg·L^−1^ IAA + 0.1% activated carbon powder, 20 g·L^−1^ sugar and 7 g·L^−1^ agar, pH 5.8). The seedlings were soaked in 500 mg·L^−1^ IBA solution over 10 mins before being placed in the bottles. Activated carbon was added to simulate the natural dark environment for plant root growth. The experiment involved two light treatments: white light quality (control, WL) and red light quality (660 nm, RL), which were provided by LED tubes (Philips Co., Ltd., Shanghai, China) fixed 20 cm above the bottle with a light intensity of 50 μmol·m^−2^·s^−1^ and 16 h∙d^−1^ light cycles. Each treatment had three replications, with 10 bottles and two seedlings in each bottles. The temperature was maintained at 23 ± 2°C, and the relative humidity was kept at 65%. All the nutrition and environmental conditions were the same except the light regimes. The basal of seedlings samples were collected for the root paraffin section, immediately frozen in liquid nitrogen, then stored at −80°C for transcriptome sequencing and phytohormone assays. At 36 DAI, fresh tea cuttings were fully harvested to analyze AR morphology.

### 2.2 Adventitious root morphological measurements

The number of rooting and non-rooting seedlings was recorded to calculate the rooting rate. A root more than 2 mm was defined as rooting successfully, namely AR formation. Rooting rate = the number of rooting seedlings / the number of all seedlings. At 36 DAI, the number of roots in each sample was counted, and the maximum root length and average root length were measured using a vernier calliper (Deli Group Co., Ltd, DL91150, Ningbo, China) to analyze.

### 2.3 Adventitious root anatomical observation

Root anatomical structure of seedlings was observed by paraffin sectioning, and images were collected by microscopic OLYMPUS BX53 (Olympus Optical Co., Ltd, Japan). The step of fixation, paraffin embedding, and sectioning as follows: the basal of seedlings samples of the 1, 4, 16, 20 DAI were immersed in alcohol-benzene for 5-10 min, xylene for 5-10 min and paraffin for 1 h in turn, respectively, and then embedded in paraffin and cut into 4‐µm-thick sections by a microtome (Leica Instruments Co., LTD RM2016, Shanghai, China). The sections were treated in 40°C warm water for 20 min, and baked in the oven (Laibo Rui Instrument Equipment Co., LTD GFL-230, Tianjin, China) at 60 °C for 30 min. After putting into xylene twice for 20 min, the slices were successively put in anhydrous ethanol for 5 min twice and 75% alcohol for 5 min, and then washed under running water. Subsequently, the slices were treated in 50%, 70% and 80% alcohol for 8 s in turn, followed by safranin staining for 1 h. The slices were treated in anhydrous ethanol I for 30 s, and anhydrous ethanol II for 1 min, followed by solid green for 30 s. After dehydration in three vats of anhydrous ethanol, the slices were placed in pure xylene for 5 min. The slices were sealed by neutral gums for microscopic examination.

### 2.4 RNA sequencing and data processing

The total RNA of eight samples was extracted by the RNAprep Pure Plant Kit (Tiangen, Beijing, China) from the frozen fresh tissue, and three biological replicates were harvested for each sample. 24 transcriptome profiles were performed by Illumina 4000 at the Lc-bio Technologies (Hangzhou, China). Clean data were obtained from Lc-bio technologies after filtering out unqualified sequences. rRNA was discarded after mapping to the rRNA database. Next, full-length non-chemical (FLNC) transcripts were identified by searching for primers at both ends of the reads. The Camellia sinensis reference genome was downloaded from the Tea Plant Information Archive (TPIA), which was used for the mapping of clean reads by HISAT package.

### 2.5 Genes expression data analysis

The differentially expressed genes were using the R package (http://www.r-project.org) with |log2 (fold change)| >1 and *p* < 0.05 as cutoffs (Jing et al., 2023). All DEGs were mapped to Gene Ontology (GO) terms in the Gene Ontology database (http://www.geneontology.org), and the output of enrichment was limited to FDR < 0.05. Pathway enrichment analysis was performed using the Kyoto Encyclopedia of Genes and Genomes (KEGG) database (Kanehisa et al., 2008), and pathways with FDR-corrected *p*≤ 0.05 were considered significantly enriched in DEGs. The expression modules in **Figure 4A** were generated by STEM v1.3.7 (Ernst and Bar-Joseph, 2006) based on the FPKM values. The hierarchical clustering of the gene sets was obtained *via* Morpheus (https://software.broadinstitute.org/morpheus/) with one minus Pearson correlation as the distance metric and average as the linkage method based on the log2 (FPKM + 1) value. The gene co-expression networks were constructed *via* the WGCNA (v 1.63) package in R (Langfelder and Horvath, 2008). The 8,860 genes with quartile 1 (Q1) expression and FPKM value greater than three were screened for co-expression network analysis based on the absolute median difference. The blockwiseModules function in the WGCNA package was used for one-step co-expression network construction, with the parameters were set as follows: minModuleSize = 100, power = 7 (0.8 was used as the correlation coefficient threshold), and TOMType = “unsigned”, mergeCutHeight = 0.25 (to merge possible similar modules), verbose = 3, and maxBlockSize = 10,000. A total of 8860 genes were clustered into 14 tissue-specific modules, and 67 genes were outliers (grey module). The eigengene value was calculated for each module and used to test the association with the phytohormone phenotype data. The networks were visualized using Cytoscape (V3.2.1) (Shannon et al., 2003).

### 2.6 Extraction and measurement of phytohormones in base of cuttings

The levels of 3-indoleacetic acid (IAA), ABA, zeatin (ZT) and gibberellic acid 3 (GA_3_) were quantified using liquid chromatogram (Agilent 1100, Agilent Technologies, Santa Clara, CA, United States). First, basal portions of seedlings at 1, 4, 16 and 20 DAI were accurately weighed to 200 mg and homogenized with 2 mL of pre-chilled 80% methanol, respectively. The mixed samples were stored at 4°C overnight and then centrifuged at 5000 r.min^-1^ for 10 min at 4°C to obtain the supernatant, which was subsequently dried with nitrogen at 4°. After continuing to decolorize by adding 2 mL of petroleum ether three times, the aqueous phase was extracted three times by ethyl acetate and blown with nitrogen to dryness at 4°C. Acetic acid solution (pH=3.5) was added and purified with a small C18 column, and the eluate was collected after elution with methanol. The system was dried under reduced pressure at 4°C and dissolved with mobile phase to a constant volume. A filter membrane of 0.22 μL or more was used for the test. Detector: UVD chromatographic column; C18 (250 × 4.6 mm; 5 μL); Injection volume, 10 μL; Wavelength, 254 nm; Flow rate, 1.0 mL·min^−1^; Mobile phase, methanol: acetic acid aqueous solution = 20:80 (pH=3.6).

### 2.7 Statistical analysis

A Shapiro-Wilk test was used to examine the normality of variables, and the mean centering or log transformation was applied to ensure normality when the data were not normally distributed. Levene’s test was used to determine the homogeneity of variances. Statistical analysis was performed using SPSS 20.0 software (SPSS Inc., Chicago, IL, USA). Independent sample T-test and one-way analysis of variance (one-way ANOVA) were used to compare study variables between red and white light. Comparison between means was determined using Duncan’s multiple intervals, with a significance level of *p* < 0.05. Two‐way analysis of variance (Two‐way ANOVA) was performed to evaluate the effects of light, induce time, and their interactions (light × induce time) on phytohormones in seedlings. Figures were generated in Excel (Excel for Mac 2011, Microsoft Corporation) and R software (version 4.2.0).

## 3 Results

### 3.1 Effect of red light on adventitious root formation and characteristics

**Figure 1:**
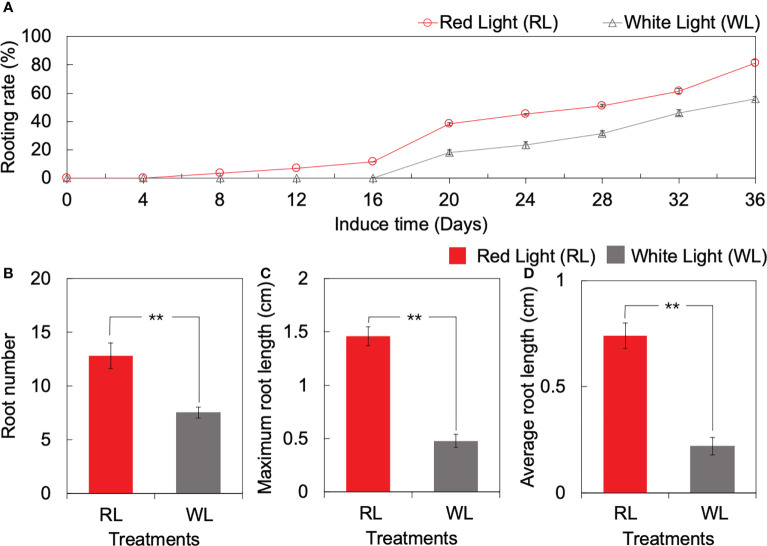
Root formation and development of tea tissue culture seeding between red light (RL) and white light (WL). **(A)** Dynamic changes of rooting rate over culturing. **(B)** Root number. **(C)** Maximum root length (unit: cm). **(D)** Average root hair length (unit: cm). ** indicates significant differences at *p* < 0.01 based on independent sample T-test.

The forming day of AR was different between RL and WL. Under RL, AR formed start at 4 DAI, and the rooting rate increased from 0% to 38.3% during the period of 4 to 20 DAI. On the contrary, the rooting rate under WL remained at 0% initially and increased gradually until 16 DAI, reaching 18.3% at 20 DAI (**Figure 1A**). Subsequently, the rooting rate showed a consistent rising trend with induction time, in which the rate under RL was higher than that under WL (**Figure 1A**). At the end of the culture period (36 DAI), the rooting rate under RL and WL reached 81.7% and 56.6%, respectively (**Figure 1A**). OOver the 36 DAI growth, the root number, maximum root length, and average root length under RL were 0.71-fold, 2.04-fold, and 2.34-fold higher than those under WL, respectively (*p* < 0.01) (**Figure 1B**). Based on the results of AR formation and development qualities, the 1, 4, 16 and 20 DAI were selected as important time points for the subsequent research.

### 3.2 Effect of red light on adventitious root morphological characteristics

**Figure 2:**
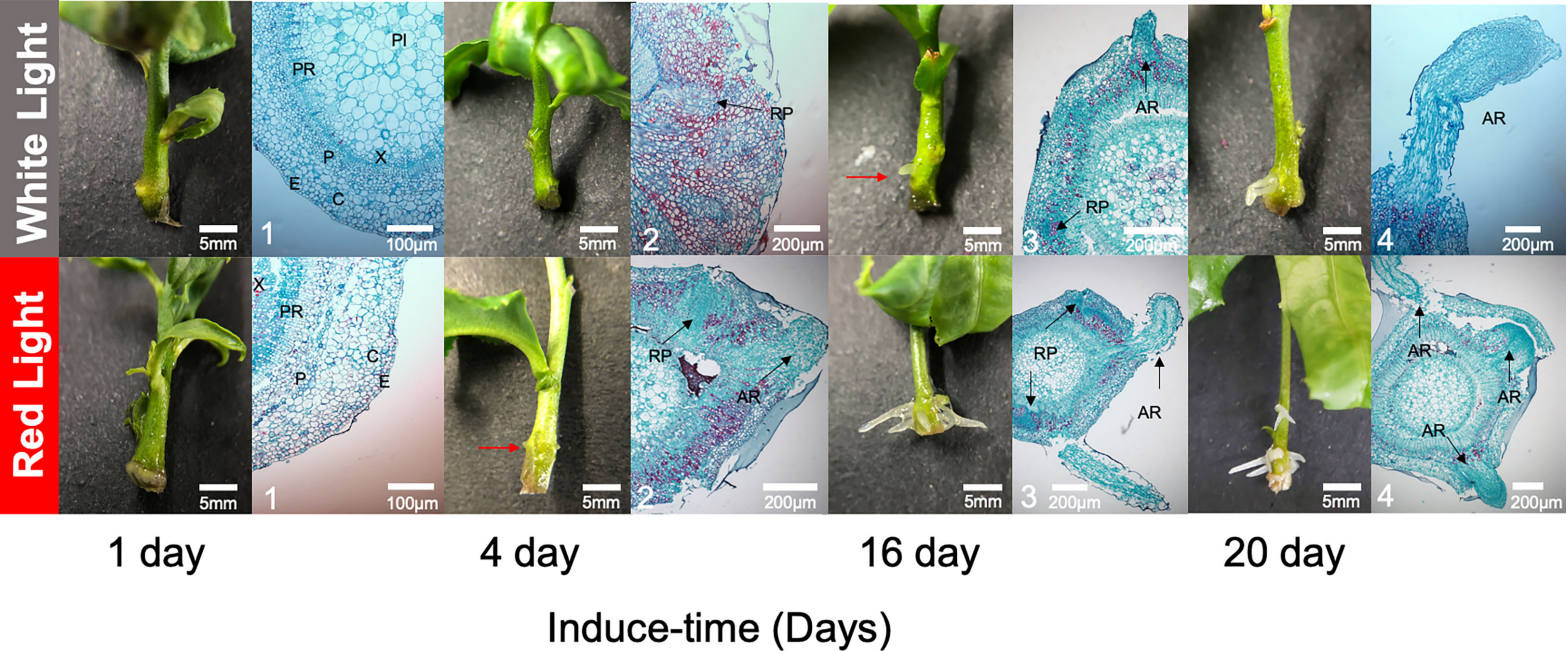
Dynamic developments of root morphological and anatomical structure between red light and white light over culturing at 1, 4, 16 and 20 days after induced treatment (DAI). The red arrow was pointed to the tiny adventitious root. The black arrows were pointed to the developing root primordia and adventitious roots. E, epidermis; C, cortex; P, phloem; PI, pith; X, xylem; PR, pith ray; RP, roots primordium; AR, adventitious root; VC, vascular cambium.

To characterize AR development, anatomical structures were observed during the inducing process. At 1 DAI, there was no difference at the surface of basal sections of seedlings between RL and WL, and the cross-section of the seedling was well organized, consisting of the epidermis (E), cortex (C), phloem (PI), vascular cambium (VC), xylem (X) and pith ray (PR) (**Figure 2**). As the induction time increased, a small white dot was obviously observed on the surface of basal sections of seedlings at 4 DAI under RL, but not under WL. Anatomical observations recorded that root primordium (RP) had developed from VC under RL at the 4 DAI and then broken through the E to be a small white dot, while the RP remained immature under WL (**Figure 2**). At the 16 DAI, AR was observed on seedlings under RL and WL, but the number and length of AR were higher under RL. At the 20 DAI, anatomical observation revealed that AR entered the elongation phase under RL and WL (**Figure 2**). There was distinct stratification during the elongation period. Among them, the most apical part of the AR consisted of a few layers of root crown cells, and further inward was the apical meristem, which was closely arranged and had a large nucleus (**Figure 2**).

### 3.3 Global analysis of the transcriptomes of seedlings

To uncover the molecular mechanisms underlying AR development, RNA-seq was performed based on deep transcriptome sequencing analysis at 1, 4, 16 and 20 DAI. Our sequencing produced over 4 GB data per sample, with Q30 (high sequencing quality) scores exceeding 97% (**Table S1**). A total of 51, 576, 462 clean reads were obtained for the RL1; 43, 623, 812 for RL4; 49, 643, 734 for RL16; 43, 205, 852 for RL20; 39, 839, 074 for WL1; 45, 828, 916 for WL4; 48, 861, 494 for WL16; and 49, 395, 078 for WL20. The reads were assembled into 53, 512 unigenes (**Table S2**). and over 85% of the reads mapped to the exon region (**Figure S1**). Out of the assembled unigenes, 21,961 (41.04%), 1,482 (2.77%) were annotated using the Gene Ontology (GO), Kyoto Encyclopedia of Genes Genomes (KEGG) (**Table S2**), indicating the high quality of our sequencing data for further analysis.

**Figure 3:**
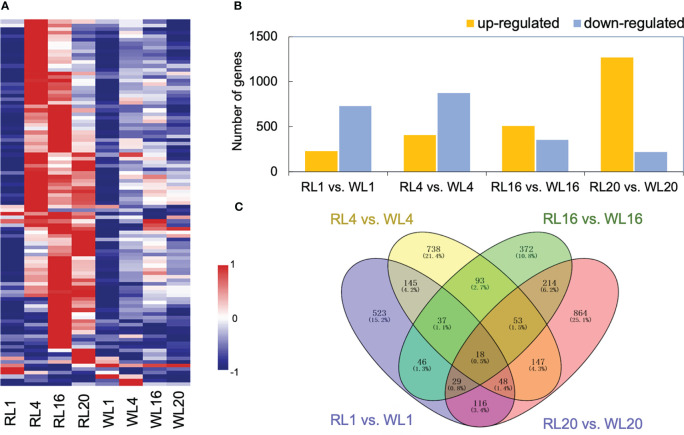
Global analysis of the transcriptomes. **(A)** Heatmap showing the different expression patterns of genes under red light (RL) and white light (WL). **(B)** Histogram showing the number of DEGs in the comparison group. **(C)** Venn diagrams showing the intersections among the four comparison groups. RL, red light; WL, white light; and the numbers were days.

The heatmap depicted that gene expression patterns were affected by light quality, showing differences between RL and WL (**Figure 3A**). At 1 DAI, there was no difference in gene expression pattern between RL and WL. However, at 4, 16 and 20 DAI, most of the DEGs were up-regulated under RL. The total number of significantly regulated genes varied across different comparisons (**Figure 3B**). The largest difference was found in WL20 vs. RL20 (1268 upregulated/221 downregulated), followed by WL4 vs. RL4 comparison (406 up-regulated/873 down-regulated). Volcano plots (**Figure S2A**) showed that the number of up-and down-regulated genes had a distinct distribution pattern between different comparisons, and the distribution pattern of down-regulated genes of WL20 vs. RL20 was higher than those of the other groups. Venn diagrams illustrated the DEGs in all induce time points and the corresponding number of DEGs. Among all comparisons, RL16 vs. WL16 and RL20 vs. WL20 shared the highest number of differential genes (314), while RL1 vs. WL1 and RL16 vs. WL16 recorded the lowest number (113) (**Figure 3C**). More, we also compared the differentially expressed genes at each developmental stage under different light qualities,including RL20 vs. RL1, RL16 vs. RL1, RL4 vs. RL1, WL20 vs. WL1, WL16 vs. WL1 and WL4 vs. WL1 (**Figure S2B**). The results showed that the RL16 vs. RL1 group had the most differential genes (5726) and the WL4 vs. WL1 group had the least group (2904). The sum of the number of DEGs in all comparison groups reached 10,575.

**Figure 4:**
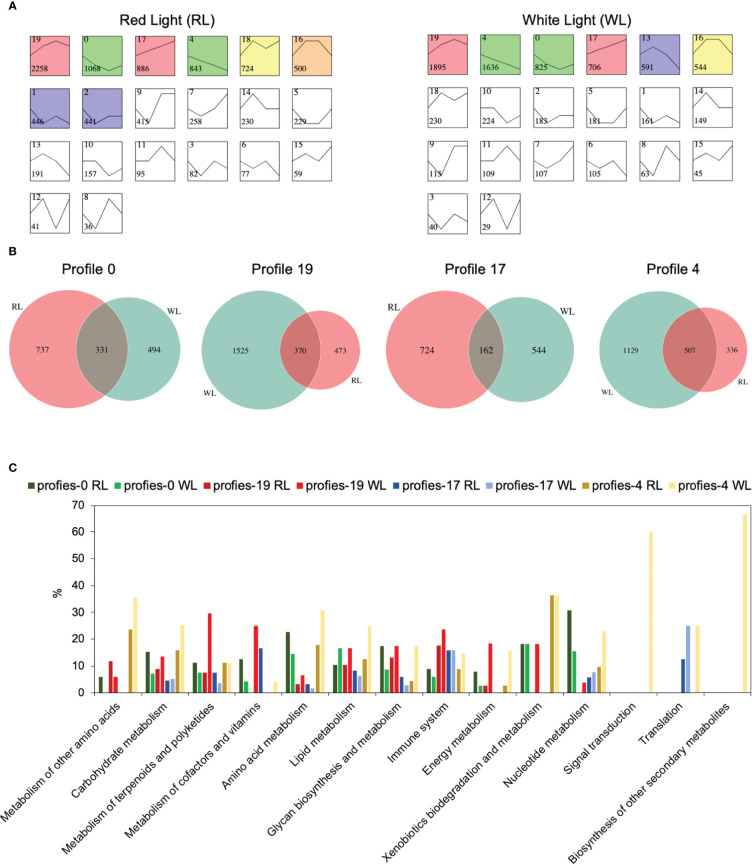
Gene expression profile analysis and functional enrichment analysis in red light (RL) and white light (WL). **(A)** Module profiles of the genes in two light qualities across four AR developmental stages. **(B)** Venn diagrams showing the number of shared and uniquely expressed genes between two light qualities in profiles 0,19,17 and 4. **(C)** KEGG pathway enrichment analysis results for the genes clustered in profiles 0,19,17 and 4 between two light qualities. The x-axis represents the pathways, and the y-axis represents the percentage of the number of enriched genes relative to the total number of genes in the pathway.

To identify DEGs profiles across AR development in each induce-time, Short Time-series Expression Miner (STEM) (Ernst and Bar-Joseph, 2006) was used to perform clustering. The results showed that RL and WL induced eight and six statistically significant model profiles (colored profiles), respectively (**Figure 4A**). In RL vs WL group, four special model profiles (profile 4, down-regulated profile; profile 17, up-regulated profile; profile 19, up-down-regulated profile; profile 0, down-up- regulated profiles) were identified. Venn diagrams revealed that the number of unique expressed genes was more than that of commonly expressed genes among the RL and WL in profile 0, profile 19 and profile 17 (**Figure 4B**), suggesting that different regulation networks were involved in AR development under two different light qualities. Gene Ontology (GO) term enrichment analysis also provided similar results (**Figure S3**). In addition, the eight gene sets of profile 0, profile 19, profile 17 and profile 4 were subjected to a pathway enrichment analysis (**Figure S4**). As shown in **Figure 4C**, the percentage of genes in most pathways was higher in WL than in RL on profile 4 and profile 19. This pattern, however, was reversed in profile 0 and profile 17, where more genes were enriched in RL. These results indicated the complicated regulatory networks involved in the development of AR under the two light qualities. Co-expression network analysis among ARs development.

### 3.4 Co-expression network analysis among ARs development

**Figure 5:**
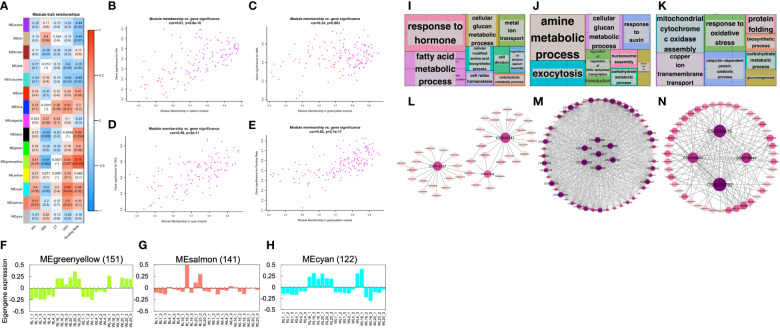
Weighted gene co-expression network analysis. **(A)** Module-phytohormone-rooting rate association. Each row corresponds to a module. Columns correspond to IAA, ABA, ZT, GA_3_ and rooting rate. The color of each cell at the row-column intersection indicates the correlation coefficient between the module and the hormone. The red-colored cell indicates a positive correlation, and the blue-colored cell means a negative correlation. The values in each cell indicate the correlation coefficient and *p*-value. **(B)** A scatterplot of Gene Significance (GS) for four traits vs. Module Membership (MM) in the highest correlation module. **(B–E)** are IAA, ABA, GA_3,_ and rooting rate, respectively. **(F–H)** Eigengene expression profiles and heat map show the FPKM of each gene in the greenyellow, salmon and cyan modules. The y-axis indicates the value of the module eigengene or the gene; the x-axis indicates the sample type and developmental stage. The number of genes in each module is indicated at the top. **(I–K)** GO term enrichment analysis results of the greenyellow, salmon, and cyan module genes visualized by the ‘TreeMap’ view of REVIGO. Each rectangle is a single cluster representative. The representatives are joined into ‘superclusters’ of loosely related terms, visualized with different colors. The size of the rectangles is adjusted to reflect the *p*-value. **(L–N)** Correlation networks of the greenyellow, salmon and cyan modules are visualized by Cytoscape.

In order to explore the hub genes that guide the phenotype, we conducted WGCNA. As shown in **Figure 5A**, IAA was highly correlated with the salmon module (r^2^ = 0.51, *p* = 0.01); while ABA was highly correlated with the greenyellow module (r^2^ = -0.61, *p* = 0.002); Similarly, GA_3_ was highly correlated with the cyan module (r^2^ = 0.64, *p* = 7e-04) and the rooting rate was highly correlated with the greenyellow module (r^2^ = 0.76, *p* = 2e-05). However, none of the modules were highly correlated with ZT. To investigate the relationship between gene significance (GS) and module membership (MM), two performed correlation analysis (**Figure 5**). Our results showed that GS and MM were highly correlated for IAA (**Figure 5**), ABA (**Figure 5C**), GA_3_ (**Figure 5D**), and Rooting Rate (**Figure 5E**). Interestingly, we found that genes belonging to different modules were associated with different hormone levels and rooting rates. The highest GS values for each phenotypes were associated with different modules, suggesting that the genes in different modules affected various hormones and rooting rates. Notably, the GS of the greenyellow was the highest among all the co-expression modules, indicating that the genes contained within this module may significantly impact the rooting rate and ABA pathway. The genes co-expressed in the greenyellow module were significantly enriched in 'response to hormone', 'fatty acid metabolic', and 'cellular glucan metabolic progress' (**Figures 5F, I**; **Table S3**). The genes co-expressed in the salmon module were significantly enriched in 'amine metabolic progress', 'exocytosis' and 'response to auxin' (**Figures 5G, J**; **Table S4**). The genes co-expressed in the cyan module were significantly enriched in 'mitochondrial cytochrome c oxidase assembly', 'copper ion transmembrane transport' and 'response to oxidative stress' (**Figures 5H, K**; **Table S5**). Moreover, the gene networks of the greenyellow, salmon and cyan modules were constructed by WGCNA and visualized in Cytoscape. Each node represented a gene, and the co-expression correlations were represented by the connecting lines (edges) between genes represented (**Figures 5I–K**). The genes with the most connections in the network were defined as hub genes, which may be the key regulatory genes. In the greenyellow module network (**Figure 5I**), the hub genes with the top three highest number of edges were CSS046741, CSS011937 (*PFB*), and CSS007948 (*HSF24*). Similarly, in the other module networks, the highly connected hub genes included CSS013695 (*YLS9*), CSS027698 (*ATL2*), CSS019093 (*PUB*), CSS000763 (*CML23*), CSS043415 (*LCL1*), CSS018696 (*LNK1*), and CSS009887 (*ANR*) (**Figures 5J, K**).

### 3.5 Hormone measured and related gene expression spatiotemporal distribution

**Figure 6:**
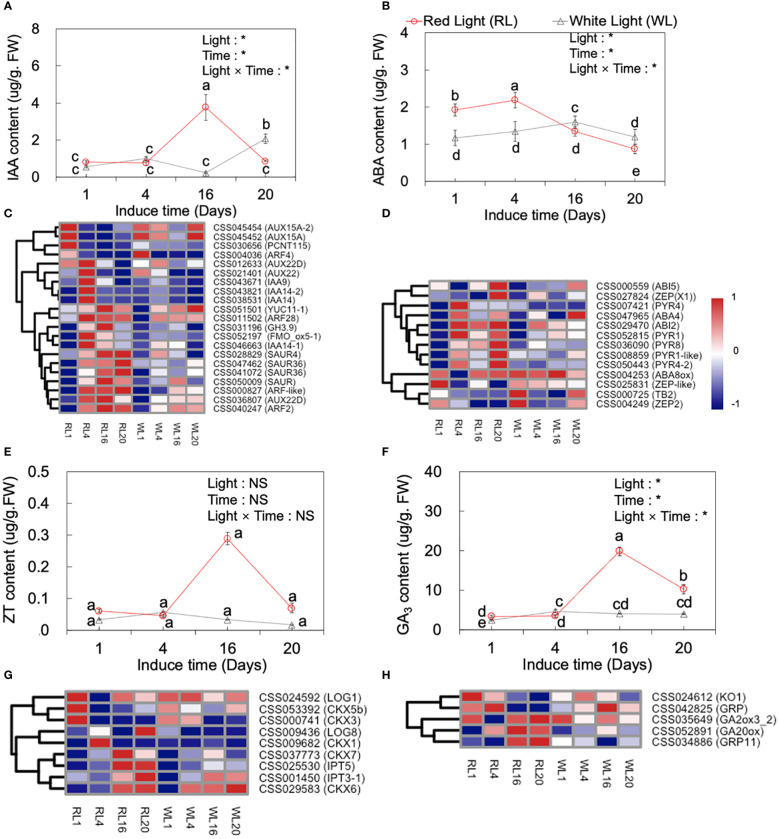
Dynamic changes of IAA **(A)**, ABA**(B)**, ZT **(E)**, and GA_3_ **(F)** content at 1, 4, 16 and 20 days after induced treatment (DAI) and the expression of major IAA- **(C)** and ABA- **(D)**, ZT- **(G)** and GA_3_- **(H)** related genes under red light (RL) and white light (WL). Lowercase letters above the bars indicate significant differences among the 1, 4, 16 and 20 DAI with *p* < 0.05. Two‐way analysis of variance (ANOVA) was performed to evaluate the effects of light, induce time, and their interactions (light × induce-time). NS means non‐significant and * indicate significant differences at *p <* 0.05. IAA, indole-3-acetic acid; ABA, abscisic acid; ZT, zeatin; GA_3_, gibberellic acid 3.

Plant phytohormones are known to play important roles in the development of AR. Previous studies have identified several plant hormones, including IAA, ABA, ZT and GA_3_, that participate in AR development (De et al., 2001). In our study, we measured the content of these hormones in both RL and WL across the four developmental stages (1, 4, 16 and 20 DAI) (**Figures 6A, B, E, F**), and hierarchical clustering analysis was performed on the log2 FPKM values for hormone-related genes (**Figures 6C, D, G, H**). Obviously, the results indicated a significant effect of light, induce time, and their interaction on IAA, ABA, ZT and GA_3_ (*p*<0.05).

As shown by **Figure 6A**, IAA hormone levels were different under RL and WL. Under RL, IAA levels increased significantly from 4 DAI to 16 DAI, reaching the highest level at 16 DAI, which was 3.74-fold higher than the average level of the 1 and 4 DAI, and then decreased by 3.34-fold at the 20 DAI, respectively (*p* < 0.05). Conversely, under WL, the highest IAA level was reached at 20 DAI (*p* < 0.05). During the induction period, a great majority of the genes showed a significant different expression pattern between RL and WL. Under RL, the relative expression of *AUX15A* (CSS045454) and PCNT115 (CSS030656) were significantly up-regulated at 1 DAI (**Figure 6C**). The *AUX22D* (CSS036807), *IAA9* (CSS043671), *IAA14* (CSS043821), and *ARF28* (CSS011502) were up-regulated at 4 DAI. Most of the following genes as *YUC11* (CSS051501), *ARF28* (CSS011502), *GH3.9* (CSS031196), *SAUR4* (CSS028829), *AUX22D* (CSS036807), *ARF2* (CSS040247), *SAUR4* (CSS028829), *SAUR36* (CSS047462), and *ARF-like* (CSS000827) maintained higher expression levels from 4 to 20 DAI under RL, which showed obvious different expression pattern compared to WL (**Figure 6C**).

The trend of ZT under RL was similar to that of IAA, reaching the highest value at 16 DAI (**Figure 6E**), while under WL its content peaked at 4 DAI and reached the lowest at 20 DAI (*p <* 0.05). Meanwhile, nine cytokinin metabolism related genes were analyzed, including LONEKY GUY (*LOG*), isopentenyl transferase (*IPT*) gene, and putative cytokinin oxidase/dehydrogenase (*CKX*) genes. Under RL, the *LOG1* (CSS024592), *CKX5b* (CSS053392) and *CKX3* (CSS000741) genes had a high expression at 1 DAI. *CKX1* (CSS009682) and *LOG8* (CSS009436) were up-regulated at 4 and 20 DAI, respectively under RL. *IPT5* (CSS025530) and *CKX6* (CSS029583) were up-regulated at 16 and 20 DAI. Taken together, these findings suggested that red light can regulate genes related to ZT synthesis to intervene in the formation and development of AR (**Figure 6G**).

The pattern of GA_3_ content under RL and WL was similar to that of ZT, with the highest level recorded at the 16 DAI under RL, which was 4.75-fold higher than the average level of 1 and 4 DAI, and subsequently decreased by 0.93-fold at 20 DAI (**Figure 6F**), (*p <* 0.05). While, under WL, the highest level of GA_3_ was also recorded at 4 DAI. The genes, involved in GA_3_ metabolism pathways, including ent-kaurene oxidase (*KO1*), gibberellin-regulated protein (*GRP*), and gibberellin 2-oxidases (*GA20ox*) showed higher expression levels under RL at 16 and 20 DAII (**Figure 6H**).

ABA content showed an earlier peak compared to other hormones, with the highest level recorded at 4 DAI and a subsequent significant decrease at 16 and 20 DAI (*p <* 0.05; **Figure 6B**). Notably, under RL, the highest level of ABA was 0.37-fold higher than that under WL. The heatmap analysis showed RL regulated genes involved in the ABA signaling pathway (**Figure 6D**). These genes included *PYR8*, *PYR1*, *PYR4* (abscisic acid receptor), *ZEP* (zeaxanthin epoxidase), *ABA8ox* (ABA 8'-hydroxylase, 2), and *ABI5* (ABSCISIC ACID-INSENSITIVE 5). Most of these genes, such as *PYR1* (CSS052815), *ABI7*(CSS007057), *PYR8* (CSS036090), *PYR4* (CSS007421), *ZEP* (CSS042660), *PYR1-like* (CSS008859), and *PYR4-2* (CSS008859), were highly expressed at 4 DAI with no significant difference in their expression levels between RL and WL after 4 DAI (**Figure 6D**). In addition, the genes *ABA8ox* (CSS004249) and *TB2* (CSS000725) expressed higher at 1 and 20 DAI under WL.

### 3.6 Analysis of TFs in DEGs under red light and white light

**Figure 7:**
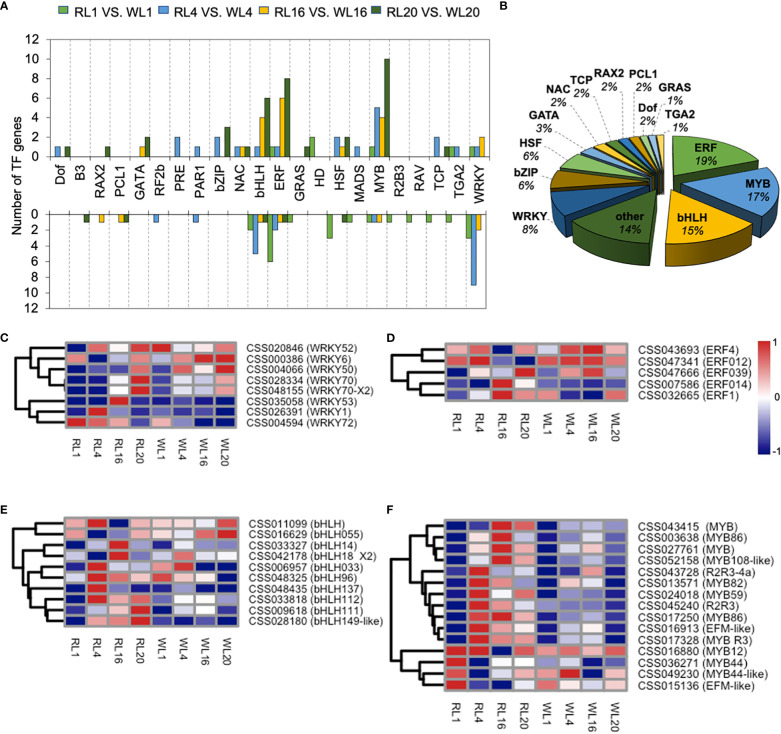
Transcription factor expression analysis. **(A)** Transcription results of transcription factors in DEGs among different comparison groups under red light (RL) and white light (WL). **(B)** The proportion of various transcription factors in DEGs. **(C)** *WRKY* expression pattern in DEGs. **(D)** *ERF* expression pattern in DEGs **(E)** *bHLH* expression pattern in DEGs. **(F)** *MYB* expression pattern in DEGs. RL, red light; WL, white light; and the numbers were days.

147 TFs of DEGs were identified in the DEGs, and we further analyzed these TFs as follows. The TFs were distributed in 22 families, and their transcriptional changes varied during the induction period. For example, the number of up-regulated genes in *MYB* family were much higher than those of down-regulated genes. In contrast, the number of down-regulated genes were higher in the *WRKY* family (**Figure 7A**). The *MYB*, *bHLH*, *ERF*, *WRKY* and *bZIP* families were the most abundant among the TFsas shown in **Figure 7B**. We further analyzed the key TFs families as heatmaps. In the *WRKY* family, most members were up-regulated by WL except of CSS004594, CSS026391 and CSS035058 (**Figure 7C**). In the *ERF* family, two members (CSS07586 and CSS032665) were up-regulated at 16 DAI under RL (**Figure 7D**). In the *bHLH* family, there were five, two and three up-regulated members at 4, 16 and 20 DAI, respectively (**Figure 7E**). Fifteen members of *MYB* were significantly up-regulated by RL, which showed a distinct pattern of expression compared to WL. Notably, the 4 DAI contained the most up-regulated genes (nine genes) (**Figure 7F**).

## 4 Discussion

### 4.1 Red light affected adventitious root development by regulating root primordia formation

AR formation is a complex process that originates from root primordia (Roycewicz and Malamy, 2014). In this study, electron microscopy was used to observe AR formation at 1, 4, 16 and 20 DAI (**Figure 2**). At the onset of light-induced treatment in *Camellia gymnogyna Chang*, PR was not observed, and AR formation mainly occurred in the VC. Compared with white light, red light had a positive effect on promoting AR formation and development at cambial cells in a similar way as that observed in *P. contorta* (Lindroth et al., 2001), echoing the promoting potential of root characteristics such as the number of roots, the maximum root length and average root length (**Figure 1**). The anatomical results further confirmed that the red light stimulated the seedlings to initiate the program of root primordia earlier from the vascular cambium, leading to earlier root formation and earlier entry into the elongation stage (**Figure 2**). It is worth noting that AR formation under RL occurred earlier than under WL, with 4 DAI being a key marker time. This may demonstrate that, as a light signal, red light could enable the cells near the vascular cambium to change undergo morphological for recovering differentiation ability (Heyl et al., 2008), which was an important prerequisite for being a root primordial protocell. Previous studies in *Picea abies* also confirmed this positive effect of red light on root development (Alallaq et al., 2020).

### 4.2 Red light affected adventitious root formation and development by regulating multiple hormones

Plant phytohormones participate in AR developments (De et al., 2001). In this study, the phytohormone levels of IAA, ABA, ZT and GA3 were significantly affected by red light (**Figure 6**). These findings were consistent with previous studies that have shown that red light can regulate the types and amounts of phytohormones in the root primordium to support AR formation (Symons and Reid, 2003; Weller et al., 2009; Chen et al., 2014; Alallaq et al., 2020). Our results further demonstrated that the effects of light, time and their interaction were significant for IAA, ABA, ZT, and GA3 (**Figure 6**).

Red light significantly affected the levels of IAA, ABA, ZT and GA_3_, and rapid increase in their content suggested their important roles in AR development. In general, the trend curves of IAA, GA_3_ and ZT in the four stages of AR are similar under red light: the level remained relatively flat from 1 to 4 DAI, and rose after 4 DAI and reached an inflection point as the highest level at 16 DAI, and then decrease after that. This trend was consistent with the anatomical data, which showed that 4 to 16 DAI was the critical periods of root primordium induction and development under red light (**Figure 2**). The transcript levels of genes in these hormone-related pathways correlated well with endogenous levels, which further demonstrated the key role of these hormones in the regulation of AR development by red light. For example, in GA_3_ signaling pathway, transcript levels determined for *GA20ox1* and *GA20ox3* genes correlated well with endogenous GA_3_ levels. The majority of genes associated with auxin signal transduction *AUX22D, IAA9, IAA14, ARF28, GH3.9, SAUR4, YUC11* and *AUX22D* up-regulated during the AR induction phase under red light. Interestingly, ABA was the first hormone response to red light at 4 DAI, suggesting that it may initially function in the stress response to red light stimulation (Lee et al., 2005), followed by a decline that could be a result of red light signal regulation, which provided a lower ABA environment to the benefit of AR development (Kim et al., 2008).

The role of hormones in AR development has been extensively studies, but their functions can vary depending on plants species. IAA, an auxin, is well known for its role in promoting AR and lateral roots growth by integrating endogenous and exogenous signals through multiple auxin-signaling modules (del Pozo et al., 2002), (Li et al., 2018). In our study, a large number of biotin-related genes were up-regulated by red light (**Figure 6C**), which suggested that red light positively influenced the growth-related hormone pathway to accelerate AR development. Scholars also believe that IAA ultimately affects AR formation by promoting the development of the root primordium (de Almeida et al., 2015). The effect of GA on AR in woody plants is still debated, as some studies suggested that endogenous GA promotes AR formation (Rasmussen et al., 2015; Tan et al., 2019) and its synthesis may be associated with the rapidly expanding cells in the root tip (Kim et al., 2008; Lange et al., 2005), while others reported poor rooting efficiency when a key GA biosynthesis gene (*AtGA20ox1*) was overexpressed (Mauriat et al., 2014). In the present study, the expression of GA-related genes induced by red light may result in high levels of GA accumulation and promote AR formation. Therefore, the role of GA investigated in individual studies with differences in plants remains different. ABA is mainly identified as an inhibitory role in AR formation, so lower levels of ABA are justified for AR differentiation (Rowe et al., 2016; Steffens et al., 2006). Previous studies have demonstrated that red light inhibits the synthesis of ABA (Barrero et al., 2014; Oh et al., 2007) and our result also showed consistently lower levels of ABA under red light compared to white light after 4 DAI. Therefore, red light may help AR development by impeding ABA synthesis. Generally, a high ratio of auxin and low ABA hormone levels provided an advantageous condition for AR regeneration (Tang et al., 2017), In summary, the AR-promoting potential of red light in tea plants was the result of complex interactions within various hormones that were regulated by the expression of related genes.

### 4.3 Red light affected genes of adventitious root development and cellular processes

By analyzing the correlation of WGCNA with phenotypic data, several co-expressed gene sets such as cyan module, greenyellow module, salmon module were identified (**Figure 5A**), which were enriched in different biological processes. This implied an important link between these biological processes and phenotypes. It is noteworthy that 'response to hormone’, ‘response to auxin' has been demonstrated by previous studies to play a crucial function in adventitious root development (Pacurar et al., 2014; Yin et al., 2020), in line with the biological processes mainly contained hormone response, cellular processes, various metabolic processes, etc (**Figures 5F–H**). Further analysis of the gene network of these modules showed that all these hub genes were associated with hormone signaling, AR development and cellular processes (**Figures 5I–K**). According to previous studies, the genes *HSF24* (CSS007948), *HTP* (CSS029354) responded to ethylene, ABA, auxin and jasmonic acid signals (Li et al., 2019). *XTH23* (CSS008293) has been studied and confirmed to play a role in cell wall extension through wall loosening. (Bischoff et al., 2009). It was important to be noted that *PUB* (CSS019093), *ERF6* (CSS040555), *ANR* (CSS009887) may be involved in the regulatory process of adventitious root development (Jia et al., 2019; Vernie et al., 2016). Overall, these hub genes may play a vital role in the process of AR and should be further studied for functional identification and analysis.

### 4.4 TFs of *MYB*, *bHLH*, *ERF*, *WRKY* and *bZIP* were involved in the regulation of adventitious root under red light

TFs play a critical role in transferring light signaling to the response genes(Lakhina et al., 2015). Multiple TFs, such as *MYB, bHLH, ERF, WRKY* and *bZIP* families, were differently expressed during induction (**Figure 7**), which indicated their important contributions to AR developments. Our result recorded that *MYB44, R2R3-MYB, MYB82* were up-regulated by red light, which is better involved in the synthesis and transport of auxin signals (Shin et al., 2007). Previous studies have shown that MYB is mainly involved in plant secondary metabolism, cell differentiation, and anti-stress response (Cominelli et al., 2005; Dal Cin et al., 2011; Ma et al., 2021). Notably, *R2R3-MYB* has been shown to be involved in cell fate determination (Tominaga et al., 2007) and regulation of root growth (Dai et al., 2012) in many studies, which may explain the higher rooting rate under red light. Meanwhile, *AP2/ERF* was found to be up-regulated by red light at 16 and 20 DAI, which may be related to the development of the crown root in AR (de Dorlodot et al., 2007). Another reports showed that *AP2* has interaction with *WOX11* to promote crown root development by regulating gene expression involved in cytokinin signaling (Zhao et al., 2015).

The up-regulation of additional TFs by red light further supported their important roles in AR development. For example, *GARS* genes have been found preferentially expressed in tomato and lupin root (Dal Cin et al., 2011; Sbabou et al., 2010). Overexpression of a predominantly root-expressed *NAC* in wheat roots also enhances root length and biomass (Chen et al., 2018). In addition, *HSF* is involved in cell division and root growth (Behmer et al., 2005), and is predominantly expressed in the roots of some plants (Dossa et al., 2016). These studies above supported the TFs that we identified are regulated by red light and play important functions in adventitious root development. However, to gain a deeper understanding of the mechanisms underlying the effects of red light, further research is necessary, including not only comparing the index of AR development, evaluating endogenous phytohormone levels, and analyzing transcriptome levels, but also the establishment of an effective systematic approach for identifying the complexity of tea plant homeostatic mechanisms under red light. Meanwhile, metabolomic analysis and transgenic technology are also strong tools for deeper research and exploration. We will continue to obtain transcriptome information and construct a functional network model to assess the regulatory network of red light, with the ultimate goal of guiding breeding and the vitro propagation.

## 5 Conclusion

Red light was found to play a crucial role in AR formation and development of Huangshan Bitter tea. Compared to white light, red light not only promoted the forming of AR, but also significantly increased the number, maximum length, and average length of roots (*p<*0.01). Root anatomical observation showed that AR originated from VC. Analysis of phytohormone levels showed significant upregulation of IAA, ZT, GA_3_ under red light, while ABA was significantly down-regulated (*p<*0.05). Meanwhile, the high spatial and temporal resolution of transcriptome data provides vital understanding of the molecular mechanisms underlying AR development in Huangshan Bitter tea. Several highly related modules and hub genes were identified, which were mainly enriched on pathways of ‘response to hormone’, ‘cellular glucan metabolic progress’ and ‘response to auxin’. Based on the above analysis, a total of 163 key genes were identified (**Table S6**), which could play a relevant role in regulating AR formation under red light. This systematic analysis provides a comprehensive reference for understanding the role of red light in AR formation, which could be valuable for breeding and promoting excellent varieties of tea plants.

## Data availability statement

The datasets presented in this study can be found in online repositories. The names of the repository/repositories and accession number(s) can be found below: NCBI Bioproject accession number: PRJNA935116.

## Author contributions

HF mined data and carried out data analyses. HF and XW conceived and designed the analysis. QC, SY, and JD contributed to the anatomical analysis of tea plants. QG edited the manuscript. QL and DW provided plant tissues. GL and QG provided laboratory facilities, and project supervision. All authors contributed to the article and approved the submitted version.
