# Supplementary material for: Comparative transcriptome analysis of molecular mechanisms underlying adventitious root developments in Huangshan Bitter tea (Camellia gymnogyna Chang) under red light quality
Source: Front Plant Sci. 2023 Mar 21;14:1154169. doi: 10.3389/fpls.2023.1154169 (PMC10070859; doi:10.3389/fpls.2023.1154169)
Supplement: Supplementary file 1 [file DataSheet_1.pdf]

Supplementary Tables

Table S1 The RNA sequencing quality of section of seedlings in Huangshan Bitter Tea

Sample	Valid Read(No.)	Valid Read(%)	Q20%	Q30%	GC content%
RL1_1	51576462	96.71	99.99	98.19	44.50
RL1_2	33217270	96.32	99.99	97.87	45
RL1_3	46623174	96.99	99.99	98.04	44.50
RL4_1	42026226	96.55	99.99	98.05	44.50
RL4_2	43623812	96.21	99.99	97.88	45
RL4_3	37305206	96.91	99.99	97.92	45
RL16_1	49643734	95.88	99.99	98.07	44.50
RL16_2	38685942	95.83	99.99	97.78	44.50
RL16_3	43652844	96.64	99.99	97.91	44.50
RL20_1	35663674	95.90	99.99	97.97	45
RL20_2	42293792	95.84	99.99	97.73	44.50
RL20_3	43205852	96.04	99.99	97.93	45
WL_1	38604300	96.85	99.99	97.91	45
WL_2	39839074	96.00	99.99	97.79	45
WL_3	38703006	96.88	99.99	98.11	45
WL4_1	37096506	96.03	99.99	97.95	44.50
WL4_2	45828916	96.49	99.99	98.01	44.50
WL4_3	39340616	96.00	99.99	97.97	45
WL16_1	44055944	96.33	99.99	97.88	45
WL16_2	44448008	96.20	99.99	97.94	45
WL16_3	48861494	93.78	99.99	98.33	45
WL20_1	49395078	95.96	99.99	97.78	45
WL20_2	42955332	94.94	99.99	98.10	45
WL20_3	30599644	95.98	99.99	98.07	45

Table S2 Summary of de novo assembly functional annotation for section of seedlings

Item	Number
Chromosome	4097
Genes(G)	53512
Transcripts(T)	53512
Average T/G	1.00
GO Annoated	21961
KEGG Annoated	1482

Table S3 GO pathway enrichment analysis of greenyellow modules

GO_ID	GO_Term	GO_function	S gene (No.)	B gene (No.)	*p*-value
GO:0055114	oxidation-reduction process	biological_process	18	1595	0.00
GO:0015936	coenzyme A metabolic process	biological_process	2	6	0.00
GO:0004420	hydroxymethylglutaryl-CoA reductase (NADPH) activity	molecular_function	2	6	0.00
GO:0050664	oxidoreductase activity, acting on NAD(P)H, oxygen as acceptor	molecular_function	2	7	0.00
GO:0004601	oxidoreductase activity, acting on NAD(P)H, oxygen as acceptor	molecular_function	4	126	0.00
GO:0004471	malate dehydrogenase (decarboxylating) (NAD+) activity	molecular_function	2	15	0.00
GO:0016491	oxidoreductase activity	molecular_function	8	563	0.00
GO:0045735	nutrient reservoir activity	molecular_function	4	129	0.00
GO:0051087	chaperone binding	molecular_function	2	16	0.00
GO:0015267	channel activity	molecular_function	3	66	0.00
GO:0032955	regulation of division septum assembly	biological_process	1	1	0.00
GO:0005634	nucleus	cellular_component	6	394	0.00
GO:0004553	hydrolase activity, hydrolyzing O-glycosyl compounds	molecular_function	5	276	0.00
GO:0006750	glutathione biosynthetic process	biological_process	1	2	0.01
GO:0003714	transcription corepressor activity	molecular_function	1	2	0.01
GO:0004363	glutathione synthase activity	molecular_function	1	2	0.01
GO:0043565	sequence-specific DNA binding	molecular_function	4	213	0.01
GO:0005975	carbohydrate metabolic process	biological_process	6	465	0.01
GO:0004357	glutamate-cysteine ligase activity	molecular_function	1	3	0.01
GO:0051301	cell division	biological_process	1	3	0.01
GO:0042398	cellular modified amino acid biosynthetic process	biological_process	1	3	0.01

GO:0016791	phosphatase activity	molecular_function	1	4	0.02
GO:0003857	3-hydroxyacyl-CoA dehydrogenase activity	molecular_function	1	4	0.02
GO:0006098	pentose-phosphate shunt	biological_process	1	5	0.02
GO:0004616	phosphogluconate dehydrogenase (decarboxylating) activity	molecular_function	1	5	0.02
GO:0010309	acireductone dioxygenase [iron(II)-requiring] activity	molecular_function	1	5	0.02
GO:0006073	cellular glucan metabolic process	biological_process	2	55	0.02
GO:0016762	xyloglucan:xyloglucosyl transferase activity	molecular_function	2	55	0.02
GO:0005618	cell wall	cellular_component	2	55	0.02
GO:0048046	apoplast	cellular_component	2	55	0.02
GO:0003824	catalytic activity	molecular_function	5	412	0.02
GO:0004556	alpha-amylase activity	molecular_function	1	6	0.02
GO:0042578	phosphoric ester hydrolase activity	molecular_function	1	6	0.02
GO:0051287	NAD binding	molecular_function	2	72	0.03
GO:0050662	coenzyme binding	molecular_function	2	74	0.03
GO:0007264	small GTPase mediated signal transduction	biological_process	1	9	0.03
GO:0005092	GDP-dissociation inhibitor activity	molecular_function	1	9	0.03
GO:0042626	ATPase-coupled transmembrane transporter activity	molecular_function	2	84	0.04
GO:0006631	fatty acid metabolic process	biological_process	1	14	0.05
GO:0006355	regulation of transcription, DNA-templated	biological_process	7	867	0.05
GO:0010181	FMN binding	molecular_function	1	15	0.06
GO:0003854	3-beta-hydroxy-delta5-steroid dehydrogenase activity	molecular_function	1	17	0.06
GO:0055085	transmembrane transport	biological_process	6	741	0.07
GO:0006694	steroid biosynthetic process	biological_process	1	20	0.08
GO:0046872	metal ion binding	molecular_function	3	250	0.08
GO:0071949	FAD binding	molecular_function	1	23	0.09

GO:0006979	response to oxidative stress	biological_process	2	126	0.09
GO:0003993	acid phosphatase activity	molecular_function	1	27	0.10
GO:0016998	cell wall macromolecule catabolic process	biological_process	1	28	0.10
GO:0004568	chitinase activity	molecular_function	1	28	0.10
GO:0006032	chitin catabolic process	biological_process	1	28	0.10
GO:0009725	response to hormone	biological_process	1	30	0.11
GO:0003700	DNA-binding transcription factor activity	molecular_function	4	489	0.13
GO:0003779	actin binding	molecular_function	1	37	0.14
GO:0016887	ATPase activity	molecular_function	2	166	0.14
GO:0008168	methyltransferase activity	molecular_function	2	166	0.14
GO:0030244	cellulose biosynthetic process	biological_process	1	39	0.14
GO:0016760	cellulose synthase (UDP-forming) activity	molecular_function	1	39	0.14
GO:0050661	NADP binding	molecular_function	1	46	0.17
GO:0051536	iron-sulfur cluster binding	molecular_function	1	59	0.21
GO:0016758	transferase activity, transferring hexosyl groups	molecular_function	3	402	0.21
GO:0020037	heme binding	molecular_function	4	600	0.21
GO:0006511	ubiquitin-dependent protein catabolic process	biological_process	1	63	0.22
GO:0006952	defense response	biological_process	1	64	0.22
GO:0000160	phosphorelay signal transduction system	biological_process	1	66	0.23
GO:0004185	serine-type carboxypeptidase activity	molecular_function	1	76	0.26
GO:0016616	oxidoreductase activity, acting on the CH-OH group of donors, NAD or NADP as acceptor	molecular_function	1	76	0.26
GO:0006351	transcription, DNA-templated	biological_process	1	83	0.28
GO:0005509	calcium ion binding	molecular_function	2	267	0.28
GO:0003677	DNA binding	molecular_function	5	914	0.29

GO:0016020	membrane	cellular_component	5	923	0.30
GO:0016787	hydrolase activity	molecular_function	2	288	0.31
GO:0030001	metal ion transport	biological_process	1	111	0.36
GO:0006508	proteolysis	biological_process	2	332	0.38
GO:0004252	serine-type endopeptidase activity	molecular_function	1	122	0.38
GO:0016788	hydrolase activity, acting on ester bonds	molecular_function	1	123	0.39
GO:0045454	cell redox homeostasis	biological_process	1	143	0.43
GO:0022857	transmembrane transporter activity	molecular_function	1	243	0.62
GO:0016021	integral component of membrane	cellular_component	3	854	0.66
GO:0016705	oxidoreductase activity, acting on paired donors, with incorporation or reduction of molecular oxygen	molecular_function	1	458	0.84
GO:0005506	iron ion binding	molecular_function	1	496	0.86
GO:0005515	protein binding	molecular_function	7	2540	0.89
GO:0004672	protein kinase activity	molecular_function	3	2115	0.99
GO:0006468	protein phosphorylation	biological_process	3	2116	0.99
GO:0005524	ATP binding	molecular_function	3	2274	1.00
GO:0003676	nucleic acid binding	molecular_function	3	3607	1.00

Table S4 GO pathway enrichment analysis of salmon modules

GO_ID	GO_Term	GO_function	S gene (No.)	B gene (No.)	*p*-value
GO:0006355	regulation of transcription, DNA-templated	biological_process	17.00	867	0
GO:0003700	DNA-binding transcription factor activity	molecular_function	13.00	489	0
GO:0005509	calcium ion binding	molecular_function	6.00	267	0
GO:0003677	DNA binding	molecular_function	10.00	914	0

GO:0016762	xyloglucan:xyloglucosyl transferase activity	molecular_function	3.00	55	0
GO:0005618	cell wall	cellular_component	3.00	55	0
GO:0006073	cellular glucan metabolic process	biological_process	3.00	55	0
GO:0048046	apoplast	cellular_component	3.00	55	0
GO:0006887	exocytosis	biological_process	2.00	28	0
GO:0000145	exocyst	cellular_component	2.00	30	0
GO:0043565	sequence-specific DNA binding	molecular_function	4.00	213	0
GO:0004553	hydrolase activity, hydrolyzing O-glycosyl compounds	molecular_function	4.00	276	0
GO:0005975	carbohydrate metabolic process	biological_process	5.00	465	0
GO:0003830	beta-1,4-mannosylglycoprotein 4-beta-N-acetylglucosaminyltransferase activity	molecular_function	1.00	5	0
GO:0006487	protein N-linked glycosylation	biological_process	1.00	6	0
GO:0006879	cellular iron ion homeostasis	biological_process	1.00	7	0
GO:0009001	serine O-acetyltransferase activity	molecular_function	1.00	8	0
GO:0006535	cysteine biosynthetic process from serine	biological_process	1.00	8	0
GO:0008199	ferric iron binding	molecular_function	1.00	9	0
GO:0004402	histone acetyltransferase activity	molecular_function	1.00	9	0
GO:0004664	prephenate dehydratase activity	molecular_function	1.00	10	0
GO:0008131	primary amine oxidase activity	molecular_function	1.00	10	0
GO:0009094	L-phenylalanine biosynthetic process	biological_process	1.00	10	0
GO:0009308	amine metabolic process	biological_process	1.00	10	0
GO:0009733	response to auxin	biological_process	2.00	90	0
GO:0061630	ubiquitin protein ligase activity	molecular_function	1.00	17	0
GO:0003712	transcription coregulator activity	molecular_function	1.00	25	0
GO:0048038	quinone binding	molecular_function	1.00	25	0
GO:0004568	chitinase activity	molecular_function	1.00	28	0

GO:0016998	cell wall macromolecule catabolic process	biological_process	1.00	28	0
GO:0006334	nucleosome assembly	biological_process	1.00	28	0
GO:0006032	chitin catabolic process	biological_process	1.00	28	0
GO:0016702	oxidoreductase activity, acting on single donors with incorporation of molecular oxygen, incorporation of two atoms of oxygen	molecular_function	1.00	29	0
GO:0003682	chromatin binding	molecular_function	1.00	30	0
GO:0005516	calmodulin binding	molecular_function	1.00	30	0
GO:0008146	sulfotransferase activity	molecular_function	1.00	45	0
GO:0003824	catalytic activity	molecular_function	3.00	412	0
GO:0015035	protein disulfide oxidoreductase activity	molecular_function	1.00	59	0
GO:0006952	defense response	biological_process	1.00	64	0
GO:0046872	metal ion binding	molecular_function	2.00	250	0
GO:0050662	coenzyme binding	molecular_function	1.00	74	0
GO:0016567	protein ubiquitination	biological_process	1.00	74	0
GO:0004842	ubiquitin-protein transferase activity	molecular_function	1.00	87	0
GO:0005507	copper ion binding	molecular_function	1.00	93	0
GO:0005737	cytoplasm	cellular_component	1.00	102	0
GO:0030001	metal ion transport	biological_process	1.00	111	0
GO:0016788	hydrolase activity, acting on ester bonds	molecular_function	1.00	123	0
GO:0005634	nucleus	cellular_component	2.00	394	0
GO:0045454	cell redox homeostasis	biological_process	1.00	143	0
GO:0009055	electron transfer activity	molecular_function	1.00	171	0
GO:0007165	signal transduction	biological_process	1.00	193	0
GO:0003924	GTPase activity	molecular_function	1.00	193	0
GO:0005525	GTP binding	molecular_function	1.00	242	1

GO:0022857	transmembrane transporter activity	molecular_function	1.00	243	1
GO:0005515	protein binding	molecular_function	8.00	2540	1
GO:0016787	hydrolase activity	molecular_function	1.00	288	1
GO:0016020	membrane	cellular_component	2.00	923	1
GO:0046983	protein dimerization activity	molecular_function	1.00	550	1
GO:0055085	transmembrane transport	biological_process	1.00	741	1
GO:0016021	integral component of membrane	cellular_component	1.00	854	1
GO:0055114	oxidation-reduction process	biological_process	2.00	1595	1
GO:0004672	protein kinase activity	molecular_function	3.00	2115	1
GO:0006468	protein phosphorylation	biological_process	3.00	2116	1
GO:0008270	zinc ion binding	molecular_function	3.00	3098	1
GO:0005524	ATP binding	molecular_function	1.00	2274	1
GO:0003676	nucleic acid binding	molecular_function	1.00	3607	1

Table S5 GO pathway enrichment analysis of cyan modules

GO_ID	GO_Term	GO_function	S gene (No.)	B gene (No.)	*p*-value
GO:0004185	serine-type carboxypeptidase activity	molecular_function	3	76	0.00
GO:0016787	hydrolase activity	molecular_function	5	288	0.00
GO:0004788	thiamine diphosphokinase activity	molecular_function	1	1	0.00
GO:0009229	thiamine diphosphate biosynthetic process	biological_process	1	1	0.00
GO:0033617	mitochondrial respiratory chain complex IV assembly	biological_process	1	1	0.00
GO:0030975	thiamine binding	molecular_function	1	1	0.00
GO:0004425	indole-3-glycerol-phosphate synthase activity	molecular_function	1	1	0.00
GO:0006817	phosphate ion transport	biological_process	1	3	0.01

GO:0005315	inorganic phosphate transmembrane transporter activity	molecular_function	1	3	0.01
GO:0004612	phosphoenolpyruvate carboxykinase (ATP) activity	molecular_function	1	3	0.01
GO:0000160	phosphorelay signal transduction system	biological_process	2	66	0.02
GO:0006094	gluconeogenesis	biological_process	1	6	0.02
GO:0005739	mitochondrion	cellular_component	1	8	0.02
GO:0030247	polysaccharide binding	molecular_function	2	86	0.03
GO:0006415	translational termination	biological_process	1	9	0.03
GO:0003747	translation release factor activity	molecular_function	1	9	0.03
GO:0005375	copper ion transmembrane transporter activity	molecular_function	1	9	0.03
GO:0035434	copper ion transmembrane transport	biological_process	1	9	0.03
GO:0016021	integral component of membrane	cellular_component	6	854	0.04
GO:0031625	ubiquitin protein ligase binding	molecular_function	1	13	0.04
GO:0009058	biosynthetic process	biological_process	2	110	0.04
GO:0016226	iron-sulfur cluster assembly	biological_process	1	15	0.04
GO:0004190	aspartic-type endopeptidase activity	molecular_function	1	15	0.04
GO:0005783	endoplasmic reticulum	cellular_component	1	19	0.05
GO:0045735	nutrient reservoir activity	molecular_function	2	129	0.05
GO:0006508	proteolysis	biological_process	3	332	0.07
GO:0016779	nucleotidyltransferase activity	molecular_function	1	29	0.08
GO:0016887	ATPase activity	molecular_function	2	166	0.08
GO:0051082	unfolded protein binding	molecular_function	1	31	0.09
GO:0006457	protein folding	biological_process	1	32	0.09
GO:0008171	O-methyltransferase activity	molecular_function	1	61	0.16
GO:0055085	transmembrane transport	biological_process	4	741	0.16
GO:0006511	ubiquitin-dependent protein catabolic process	biological_process	1	63	0.17

GO:0015267	channel activity	molecular_function	1	66	0.17
GO:0005509	calcium ion binding	molecular_function	2	267	0.18
GO:0050662	coenzyme binding	molecular_function	1	74	0.19
GO:0005524	ATP binding	molecular_function	9	2274	0.20
GO:0016829	lyase activity	molecular_function	1	81	0.21
GO:0042626	ATPase-coupled transmembrane transporter activity	molecular_function	1	84	0.22
GO:0006979	response to oxidative stress	biological_process	1	126	0.31
GO:0004601	peroxidase activity	molecular_function	1	126	0.31
GO:0005515	protein binding	molecular_function	9	2540	0.31
GO:0003824	catalytic activity	molecular_function	2	412	0.33
GO:0008168	methyltransferase activity	molecular_function	1	166	0.38
GO:0007165	signal transduction	biological_process	1	193	0.43
GO:0043565	sequence-specific DNA binding	molecular_function	1	213	0.46
GO:0046983	protein dimerization activity	molecular_function	2	550	0.47
GO:0016747	transferase activity, transferring acyl groups other than amino-acyl groups	molecular_function	1	222	0.47
GO:0016491	oxidoreductase activity	molecular_function	2	563	0.49
GO:0055114	oxidation-reduction process	biological_process	5	1595	0.49
GO:0022857	transmembrane transporter activity	molecular_function	1	243	0.51
GO:0020037	heme binding	molecular_function	2	600	0.52
GO:0004553	hydrolase activity, hydrolyzing O-glycosyl compounds	molecular_function	1	276	0.55
GO:0005634	nucleus	cellular_component	1	394	0.68
GO:0016758	transferase activity, transferring hexosyl groups	molecular_function	1	402	0.69
GO:0006355	regulation of transcription, DNA-templated	biological_process	2	867	0.72
GO:0016705	oxidoreductase activity, acting on paired donors, with incorporation or reduction of molecular oxygen	molecular_function	1	458	0.74

GO:0004672	protein kinase activity	molecular_function	5	2115	0.74
GO:0006468	protein phosphorylation	biological_process	5	2116	0.74
GO:0005975	carbohydrate metabolic process	biological_process	1	465	0.74
GO:0016020	membrane	cellular_component	2	923	0.75
GO:0003700	DNA-binding transcription factor activity	molecular_function	1	489	0.76
GO:0005506	iron ion binding	molecular_function	1	496	0.76
GO:0003677	DNA binding	molecular_function	1	914	0.93
GO:0003676	nucleic acid binding	molecular_function	5	3607	0.98

Table S6 Total of 163 key genes by analysis

gene_id	moduleColors	ath_anno	RL4 vs RL1	RL16 vs RL1	RL20 vs RL1	WL4 vs WL1	WL16 vs WL1	WL20 vs WL1	RL1 vs WL1	RL4 vs WL4	RL16 vs WL16	RL20 vs WL20	profile0	profile4	profile16	profile19
CSS000415	cyan	AT5G11720	no	no	no	yes	no	no	no	no	no	no	no	no	no	yes
CSS000569	salmon		no	no	no	no	no	no	no	no	no	no	no	no	no	no
CSS000763	salmon		no	yes	yes	no	no	yes	no	no	yes	yes	no	no	no	yes
CSS000855	greenyellow	AT4G10380	no	yes	yes	no	yes	yes	no	no	no	no	no	no	yes	no
CSS001103	cyan	AT5G58960	no	no	no	no	no	no	no	no	no	no	no	no	no	no
CSS001105	cyan		no	no	no	no	no	no	no	no	no	yes	no	no	no	no
CSS001127	greenyellow		yes	yes	yes	yes	yes	yes	no	no	no	no	no	no	yes	no
CSS001921	salmon		no	no	no	no	no	no	no	no	no	no	no	no	no	no
CSS002052	salmon		no	yes	yes	no	no	yes	no	no	yes	no	no	no	no	no
CSS002341	salmon		no	yes	yes	no	no	yes	no	no	yes	yes	no	no	no	yes
CSS002514	salmon	AT5G46760	no	no	no	no	no	no	no	no	no	no	no	no	no	no

CSS003347	salmon	AT3G44260	yes	yes	yes	no	no	yes	yes	no	yes	yes	no	no	no	yes
CSS003433	cyan	AT1G62380	no	no	no	yes	no	no	no	no	no	no	no	no	no	no
CSS003550	greenyellow		no	yes	yes	no	no	yes	no	no	no	no	no	no	yes	no
CSS004317	greenyellow	AT4G25810	no	yes	yes	no	no	yes	yes	no	no	no	no	no	yes	no
CSS004576	salmon		no	yes	no	no	no	no	no	no	no	no	no	no	no	yes
CSS004624	cyan	AT3G07650	no	yes	yes	no	no	yes	no	no	yes	yes	no	yes	no	no
CSS005124	salmon	AT2G22500	no	no	no	no	no	no	no	no	yes	yes	no	yes	no	no
CSS005719	salmon	AT4G23660	no	yes	yes	no	no	yes	no	no	yes	no	no	no	no	yes
CSS005823	salmon	AT1G35140	no	yes	no	no	yes	no	no	no	yes	yes	yes	no	no	no
CSS005985	salmon		no	yes	yes	no	no	yes	no	no	yes	no	no	no	yes	no
CSS006224	greenyellow		no	no	no	yes	yes	no	no	no	no	no	no	no	no	yes
CSS006926	greenyellow	AT5G40340	no	no	yes	no	no	yes	no	no	no	no	no	no	yes	no
CSS006999	cyan		no	yes	yes	no	yes	yes	no	no	no	no	no	no	yes	yes
CSS007347	cyan	AT5G14610	no	no	no	no	no	no	no	no	no	no	no	no	no	no
CSS007575	greenyellow		no	yes	yes	no	no	yes	no	no	no	no	no	no	yes	no
CSS007576	greenyellow		no	yes	yes	no	no	yes	no	no	no	no	no	no	yes	no
CSS007948	greenyellow		no	yes	yes	no	no	yes	no	no	no	no	no	no	yes	no
CSS008106	salmon	AT5G57510	no	yes	yes	no	no	yes	no	no	yes	yes	no	no	no	yes
CSS008292	salmon	AT4G25810	no	yes	yes	no	no	yes	no	no	yes	no	no	no	no	no
CSS008293	salmon	AT4G25810	no	yes	no	no	no	no	no	no	yes	yes	no	no	no	no
CSS009069	salmon	AT3G12630	no	no	no	no	no	no	no	no	no	no	no	no	no	no
CSS009484	greenyellow	AT5G09620	no	yes	yes	no	no	yes	no	no	no	no	no	no	yes	no
CSS009887	cyan		no	yes	yes	yes	yes	yes	no	yes	no	yes	no	no	yes	yes
CSS010442	salmon	AT1G78230	no	yes	no	no	no	no	no	no	no	no	no	no	no	yes
CSS010705	greenyellow	AT3G23290	no	yes	yes	no	yes	yes	no	no	no	no	no	no	yes	no

CSS010783	greenyellow	AT1G19150	no	no	yes	no	yes	yes	no	no	no	no	no	no	yes	no
CSS011044	greenyellow	AT3G59920	no	no	no	no	no	no	no	no	no	no	no	no	no	no
CSS011305	greenyellow	AT3G55370	no	yes	yes	yes	yes	yes	no	no	no	no	no	no	yes	yes
CSS011866	salmon		no	yes	yes	no	no	yes	yes	no	yes	no	no	no	no	yes
CSS011891	salmon		no	no	yes	no	no	yes	no	no	yes	yes	no	no	yes	no
CSS011893	salmon	AT1G62760	no	no	no	no	no	no	no	no	no	no	no	no	no	no
CSS011937	greenyellow		no	no	yes	yes	yes	yes	no	no	no	no	no	no	yes	no
CSS012077	greenyellow		no	yes	yes	no	yes	yes	no	no	no	no	no	yes	no	no
CSS012171	greenyellow	AT4G39990	no	no	yes	yes	yes	yes	no	no	no	no	no	no	yes	no
CSS013205	greenyellow	AT3G59350	no	no	no	no	no	no	no	no	no	no	no	no	no	no
CSS013258	greenyellow	AT1G76690	no	yes	yes	no	yes	yes	no	no	no	no	no	yes	no	no
CSS013695	salmon		no	yes	no	no	no	no	no	no	yes	yes	no	no	yes	no
CSS013728	greenyellow		yes	yes	yes	yes	yes	yes	no	no	no	yes	no	no	yes	yes
CSS013909	salmon		no	no	no	no	no	no	no	no	yes	yes	no	yes	no	no
CSS014599	salmon		no	yes	yes	yes	yes	yes	no	no	no	no	no	no	yes	yes
CSS014626	greenyellow		no	yes	yes	no	no	yes	no	no	no	no	no	no	yes	no
CSS014720	cyan	AT3G63140	no	yes	yes	no	no	yes	no	no	yes	yes	no	no	no	no
CSS014847	greenyellow	AT1G64060	no	yes	yes	no	yes	yes	no	no	no	no	no	no	yes	yes
CSS015268	greenyellow	AT4G16780	no	no	yes	no	no	yes	no	no	no	no	no	no	yes	no
CSS015669	salmon	AT2G18260	no	no	no	no	no	no	no	no	no	no	no	no	no	no
CSS016526	cyan	AT1G02880	no	no	no	no	no	no	no	no	yes	no	no	no	no	no
CSS016654	greenyellow	AT3G31320	no	yes	yes	yes	yes	yes	no	no	no	no	no	no	yes	no
CSS017443	greenyellow		yes	yes	yes	yes	yes	yes	no	no	no	yes	no	no	yes	yes
CSS017896	greenyellow	AT5G58160	no	no	no	no	no	no	no	no	no	no	no	no	no	no
CSS018103	salmon		yes	yes	yes	yes	no	yes	no	no	no	no	no	no	no	yes

CSS018563	greenyellow		no	yes	yes	no	yes	yes	yes	no	no	no	no	no	yes	no
CSS018696	cyan	AT5G64170	no	yes	yes	yes	no	yes	no	no	no	yes	no	no	yes	yes
CSS018816	greenyellow		no	yes	yes	no	no	yes	no	no	no	no	no	no	yes	no
CSS019093	salmon	AT5G09800	no	no	no	no	no	no	no	no	yes	yes	no	no	no	no
CSS019151	salmon	AT4G27280	no	no	no	no	yes	no	no	yes	yes	yes	no	no	no	no
CSS019750	cyan		yes	yes	yes	no	no	yes	no	no	no	yes	no	no	no	yes
CSS020651	salmon	AT2G38470	no	no	no	no	no	no	no	no	no	no	no	no	no	no
CSS021009	cyan		no	no	yes	no	no	yes	no	no	no	no	no	no	yes	no
CSS021042	salmon	AT1G17420	no	yes	yes	no	no	yes	no	no	no	no	no	no	no	yes
CSS021084	cyan		no	yes	yes	yes	no	yes	no	no	no	no	no	no	no	no
CSS021145	greenyellow	AT2G03090	no	yes	yes	no	yes	yes	no	no	no	yes	no	yes	no	no
CSS021283	cyan	AT3G18830	no	yes	yes	yes	yes	yes	no	no	no	no	no	no	yes	yes
CSS022173	greenyellow	AT5G07390	no	yes	yes	no	yes	yes	no	no	no	no	no	no	yes	no
CSS022389	salmon	AT4G31550	no	no	no	no	no	no	no	no	no	no	no	no	no	no
CSS022714	cyan		no	yes	yes	no	no	yes	no	no	no	no	no	no	no	no
CSS023089	cyan	AT2G03120	no	no	no	no	no	no	no	no	no	no	no	no	no	no
CSS023419	salmon	AT1G34180	no	no	yes	no	no	yes	no	no	yes	yes	no	no	yes	no
CSS024004	salmon		no	no	yes	no	no	yes	yes	no	yes	no	no	no	yes	no
CSS024071	greenyellow	AT1G08110	no	no	no	no	no	no	no	no	no	no	no	no	no	no
CSS025132	greenyellow	AT4G14130	yes	yes	yes	yes	yes	yes	no	no	no	no	no	no	yes	no
CSS025381	cyan	AT3G46640	yes	yes	yes	yes	no	yes	no	no	yes	yes	yes	no	no	no
CSS025712	greenyellow	AT5G52510	no	yes	no	no	no	no	no	no	no	no	no	no	yes	no
CSS025894	cyan	AT1G25280	no	no	no	no	no	no	no	no	no	no	no	no	no	no
CSS026511	salmon		no	no	no	no	no	no	no	no	no	no	no	no	no	no
CSS027243	cyan	AT4G32530	no	no	no	no	no	no	no	no	no	no	no	no	no	no

CSS027426	greenyellow	AT4G14130	yes	yes	yes	yes	yes	yes	no	no	no	no	no	no	yes	no
CSS027501	salmon		no	yes	yes	no	no	yes	no	no	no	no	no	no	no	no
CSS027503	salmon		no	no	no	no	no	no	no	no	no	no	no	no	no	no
CSS027552	salmon	AT4G33920	no	no	no	no	no	no	no	no	no	no	no	no	no	no
CSS027582	salmon	AT3G55840	yes	yes	yes	no	no	yes	no	no	no	yes	no	no	no	no
CSS027698	salmon		no	yes	yes	no	no	yes	no	no	yes	yes	no	no	no	no
CSS028081	cyan	AT3G63410	no	no	no	no	no	no	no	no	no	no	no	no	no	no
CSS028420	salmon		no	no	no	no	no	no	no	no	yes	yes	no	yes	no	no
CSS028656	greenyellow	AT3G16330	no	yes	yes	yes	yes	yes	no	no	no	no	no	no	yes	no
CSS029354	salmon	AT3G62070	no	yes	yes	no	no	yes	no	no	yes	no	no	no	no	no
CSS029610	salmon		no	yes	yes	no	no	yes	no	no	no	no	no	no	no	no
CSS029616	greenyellow	AT5G14390	no	no	no	no	no	no	no	no	no	no	no	no	no	no
CSS029699	salmon	AT4G25810	no	yes	yes	no	no	yes	yes	no	yes	no	no	no	no	no
CSS030276	cyan	AT1G06130	no	no	no	no	no	no	no	no	no	no	no	no	no	no
CSS030597	cyan	AT1G72160	no	no	no	no	no	no	no	no	no	no	no	no	no	no
CSS031054	greenyellow	AT3G25800	no	no	no	no	no	no	no	no	no	no	no	no	no	no
CSS031066	salmon	AT1G68580	no	no	no	no	no	no	no	no	no	no	no	no	no	no
CSS031255	salmon		no	no	yes	no	no	yes	no	no	yes	no	no	no	yes	no
CSS031795	salmon	AT5G58620	yes	yes	yes	no	no	yes	no	no	no	no	no	no	no	no
CSS032388	greenyellow	AT2G28810	no	yes	yes	no	yes	yes	no	no	no	no	no	no	yes	no
CSS032805	cyan		no	yes	yes	yes	yes	yes	no	no	no	no	no	no	yes	yes
CSS032818	salmon	AT2G26660	yes	yes	yes	yes	no	yes	no	no	yes	yes	no	no	no	no
CSS033202	greenyellow		no	no	yes	no	yes	yes	no	no	no	no	no	no	yes	no
CSS034963	greenyellow			no	yes	no	yes	yes	no	no	no	no	no	no	yes	no

			no													
CSS035465	cyan	AT3G26570	no	no	no	no	no	no	no	no	no	no	no	no	no	no
CSS035512	greenyellow	AT1G08340	no	no	yes	no	yes	yes	no	no	no	no	no	no	yes	no
CSS035666	cyan		no	no	yes	yes	yes	yes	no	no	no	no	no	no	yes	yes
CSS035784	greenyellow	AT3G58710	no	no	yes	no	no	yes	no	no	no	no	no	no	yes	no
CSS036459	cyan	AT1G56300	no	yes	yes	no	no	yes	no	no	no	no	no	no	no	no
CSS036498	greenyellow	AT2G23290	no	no	yes	no	no	yes	no	no	no	no	no	no	no	no
CSS036792	cyan	AT1G01060	no	yes	yes	yes	yes	yes	no	no	no	yes	no	no	no	yes
CSS036819	cyan		no	yes	yes	yes	yes	yes	no	yes	no	yes	no	no	yes	yes
CSS037035	salmon		no	yes	yes	no	no	yes	no	no	no	no	no	no	yes	yes
CSS037186	salmon	AT1G35140	no	no	no	no	yes	no	no	no	yes	yes	yes	no	no	no
CSS037593	greenyellow		no	yes	yes	no	no	yes	no	no	no	no	no	no	yes	no
CSS038401	cyan	AT2G24270	no	no	no	no	no	no	no	no	no	no	no	no	no	no
CSS038581	greenyellow	AT4G14130	yes	yes	yes	yes	yes	yes	no	no	no	no	no	no	yes	no
CSS038826	cyan	AT4G25434	no	no	no	no	no	no	no	no	no	no	no	no	no	no
CSS040555	salmon	AT4G17490	yes	yes	yes	no	no	yes	yes	no	yes	no	no	no	no	no
CSS040622	greenyellow		no	yes	yes	yes	yes	yes	no	yes	no	no	no	no	yes	yes
CSS041345	greenyellow	AT2G36890	no	yes	yes	no	no	yes	no	no	no	no	no	no	yes	no
CSS041376	salmon	AT5G67450	yes	yes	yes	no	no	yes	no	yes	yes	yes	no	no	no	no
CSS042666	greenyellow	AT3G50360	no	no	no	no	no	no	no	no	no	no	no	no	no	no
CSS043142	cyan	AT5G20630	no	yes	yes	no	no	yes	yes	yes	no	yes	no	no	no	no
CSS043163	salmon	AT5G22250	yes	yes	yes	no	no	yes	no	no	yes	yes	no	no	no	no
CSS043415	cyan	AT5G02840	no	yes	yes	yes	yes	yes	no	no	no	yes	no	no	yes	yes
CSS043620	greenyellow	AT5G40850	no	yes	yes	no	no	yes	no	no	no	no	no	no	no	no
CSS043693	salmon	AT3G15210	no	no	no	no	no	no	no	no	yes	no	no	no	no	no

CSS043907	salmon		yes	yes	yes	no	no	yes	yes	no	yes	yes	no	no	no	yes
CSS045618	greenyellow	AT5G40330	no	no	no	no	no	no	no	no	no	no	no	no	yes	no
CSS046256	greenyellow	AT5G63180	no	yes	yes	no	yes	yes	no	no	no	no	no	yes	no	no
CSS046672	cyan		no	no	no	no	no	no	no	no	no	no	no	no	no	no
CSS046741	greenyellow		no	no	no	no	yes	no	no	no	no	no	no	no	yes	no
CSS046803	salmon	AT4G20780	no	yes	no	no	no	no	no	no	no	no	no	no	no	no
CSS047124	greenyellow	AT3G62700	no	no	no	no	no	no	no	no	no	no	no	no	no	no
CSS047142	cyan	AT3G09350	no	no	no	no	no	no	no	no	no	no	no	no	no	no
CSS047148	salmon		no	no	no	no	no	no	no	no	no	no	no	no	no	no
CSS047943	greenyellow		no	no	no	no	no	no	no	no	no	no	no	no	no	no
CSS048163	greenyellow	AT3G13810	no	no	no	no	no	no	no	no	no	no	no	no	no	no
CSS048392	greenyellow	AT1G01020	yes	yes	yes	no	yes	yes	no	no	no	no	no	no	yes	no
CSS048880	greenyellow	AT5G67520	yes	yes	yes	yes	yes	yes	no	no	no	no	no	no	yes	no
CSS048934	greenyellow	AT3G14205	no	no	no	no	no	no	no	no	no	no	no	no	no	no
CSS049902	greenyellow	AT2G19900	no	no	no	no	no	no	no	no	no	no	no	no	no	no
CSS050154	greenyellow		yes	yes	yes	yes	yes	yes	no	no	no	no	no	no	yes	yes
CSS050217	greenyellow		no	yes	yes	no	yes	yes	no	no	no	no	no	no	yes	yes
CSS050381	cyan	AT3G02470	no	yes	yes	no	no	yes	no	no	yes	yes	no	no	no	no
CSS050478	salmon		no	no	no	no	no	no	no	no	no	no	no	no	no	no
CSS050483	greenyellow		no	yes	yes	no	yes	yes	no	no	no	no	no	no	yes	no
CSS050729	cyan		no	yes	yes	no	no	yes	no	no	no	no	no	no	yes	no
CSS050774	greenyellow		no	yes	yes	no	no	yes	no	no	no	no	no	no	yes	no
CSS050848	cyan	AT4G34230	no	no	no	no	no	no	no	no	no	no	no	no	no	no
CSS051316	greenyellow	AT2G42590	no	no	no	no	no	no	no	no	no	no	no	no	no	no
CSS051410	cyan	AT4G15550	no	yes	yes	no	no	yes	no	no	no	no	no	no	yes	no

CSS052235	salmon		no	yes	yes	no	no	yes	no	no	no	no	no	no	no	no
CSS052463	cyan	AT1G05260	no	yes	yes	no	no	yes	no	no	no	no	no	no	no	no
CSS052642	cyan	AT4G15130	no	yes	yes	no	no	yes	no	no	no	no	no	yes	no	no
CSS052690	greenyellow		no	no	yes	no	no	yes	no	no	no	no	no	no	no	no
