# Supplementary material for: Comparative transcriptome analysis of molecular mechanisms underlying adventitious root developments in Huangshan Bitter tea (Camellia gymnogyna Chang) under red light quality
Source: Front Plant Sci. 2023 Mar 21;14:1154169. doi: 10.3389/fpls.2023.1154169 (PMC10070859; doi:10.3389/fpls.2023.1154169)
Supplement: Supplementary file 2 [file DataSheet_2.pdf]

Supplementary Figures

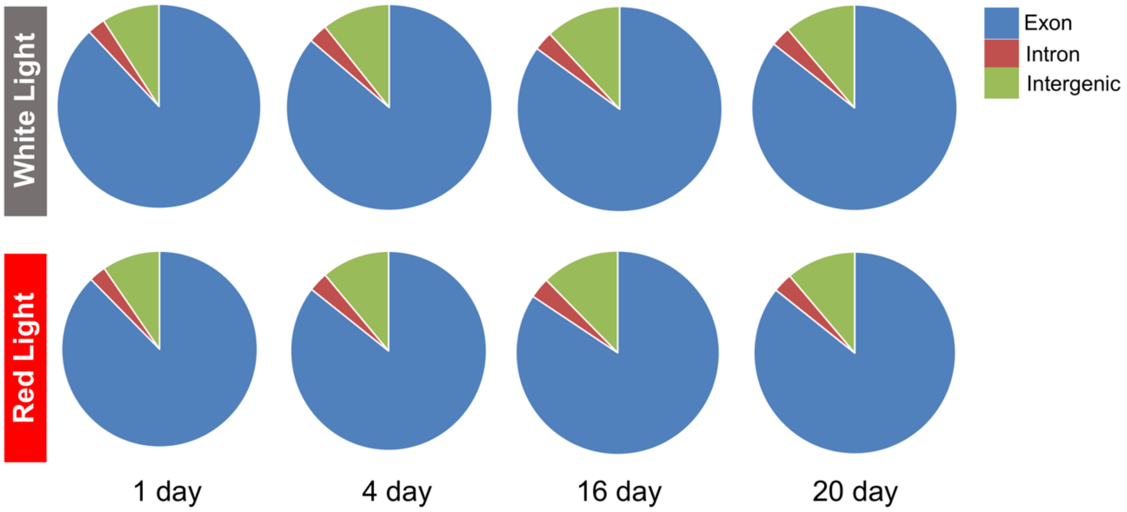

FIGURE S1 Statistic maps of gene of coverage. Blue represents exon proportion, red represents intron, and green represents intergenic. Generally, the ratio of reads compared to Exon is the highest (transcriptome sequencing refers to the mRNA transcribed from exons).

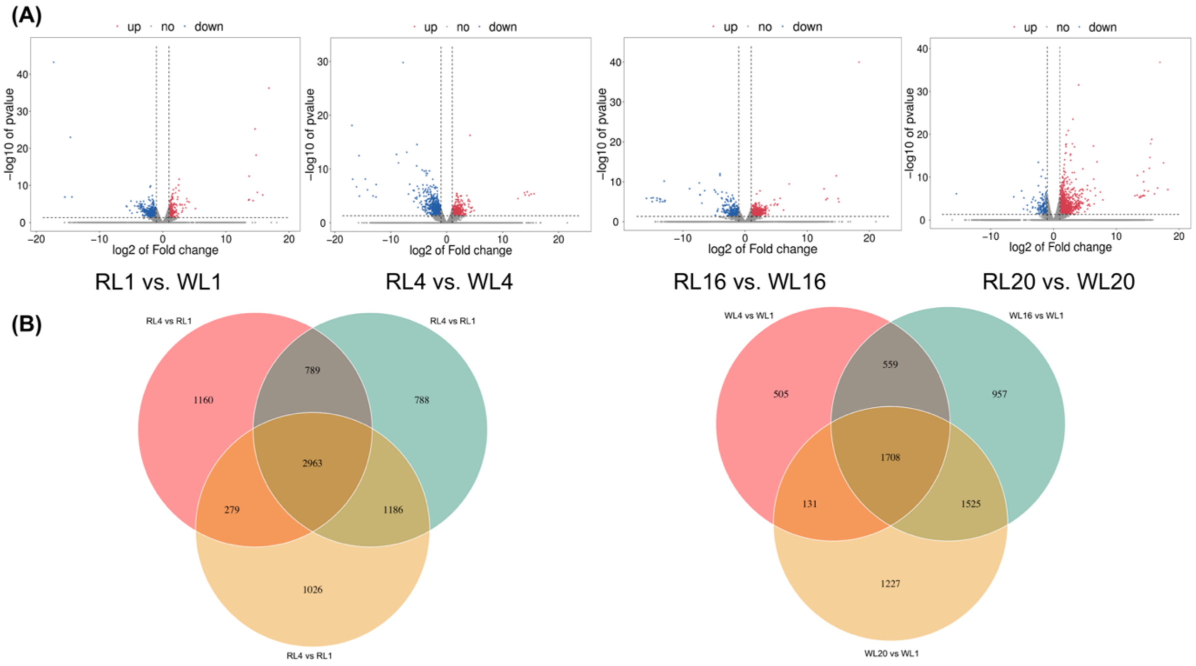

FIGURE S2 (A) Volcano plots between treatments and control. Blue and Red points represent up- and down-regulated genes, respectively. Gray points represent no difference genes. (B) Venn diagrams showing the DEGs interaction among different development stage in RL and WL respectively.

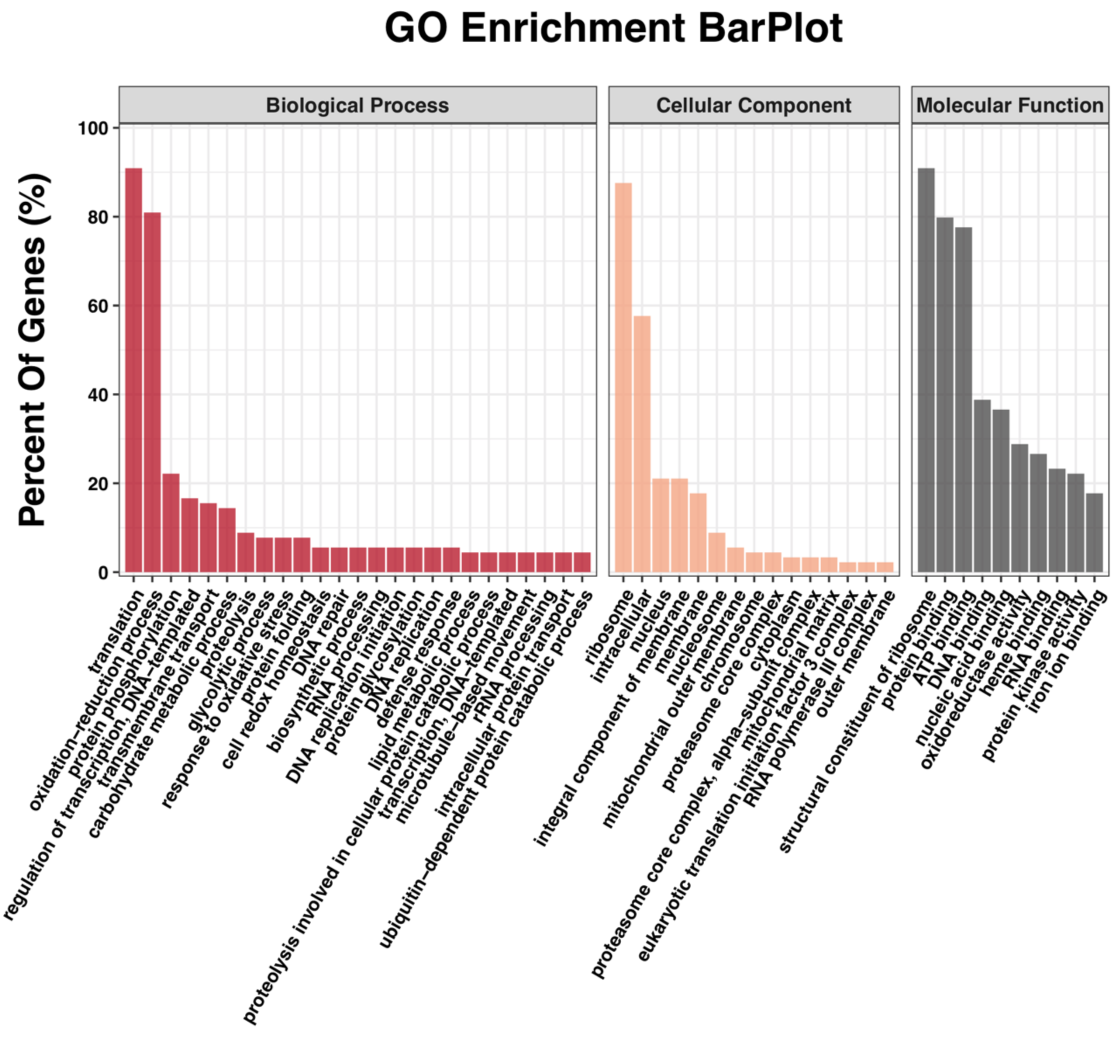

S3-A: 0 profile-red GO Term

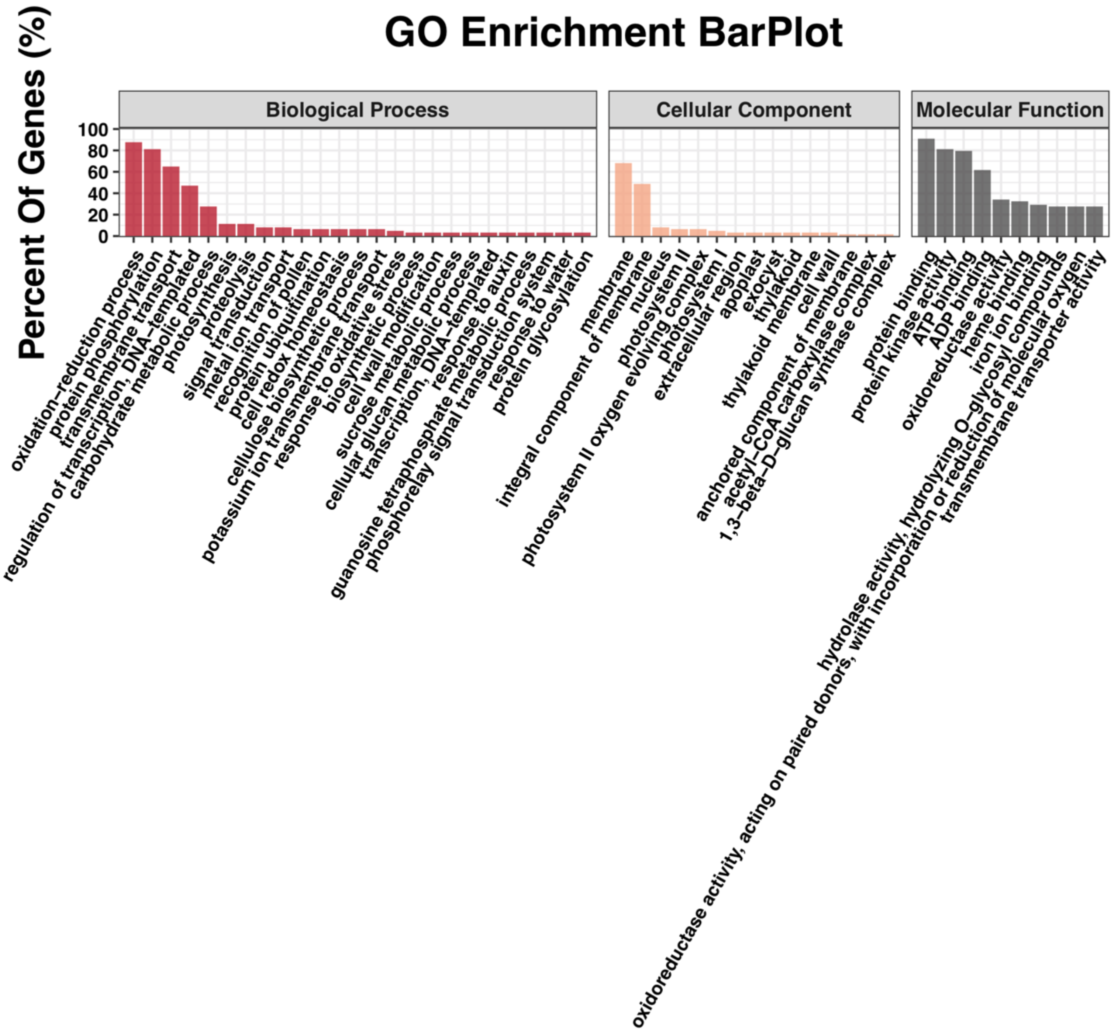

S3-B: 19 profile-red GO Term

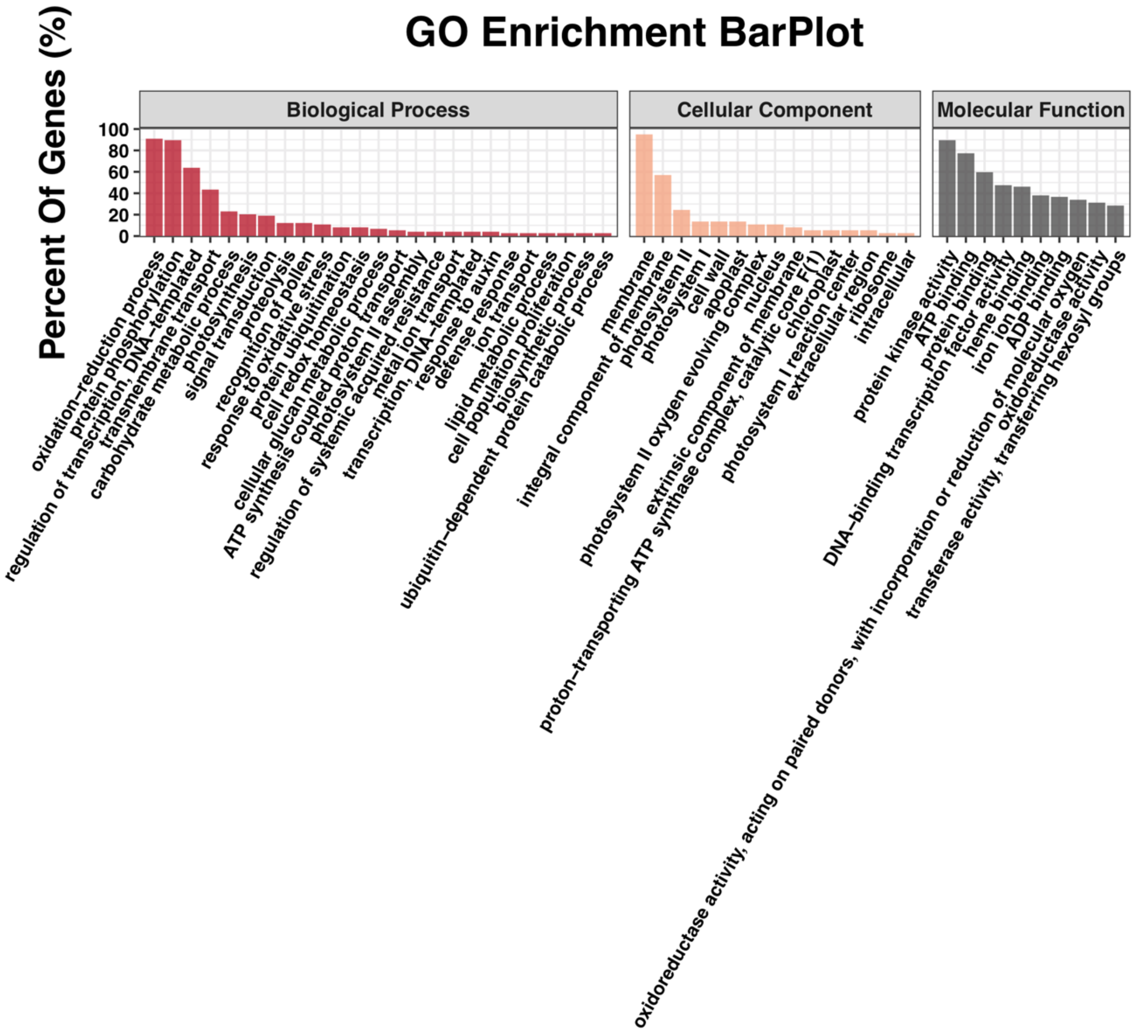

S3-C: 17 profile-red GO Term

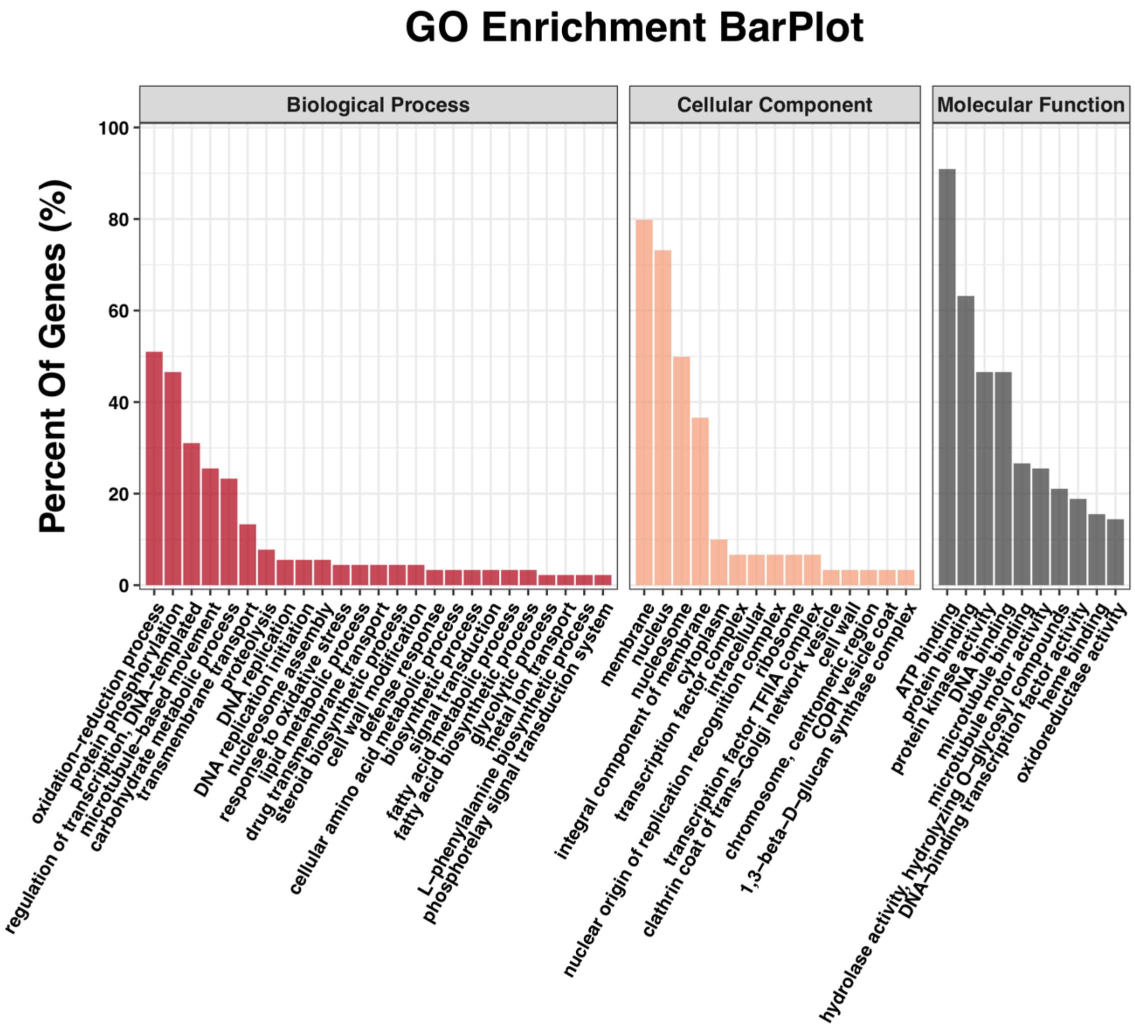

S3-D: 4 profile-red GO Term

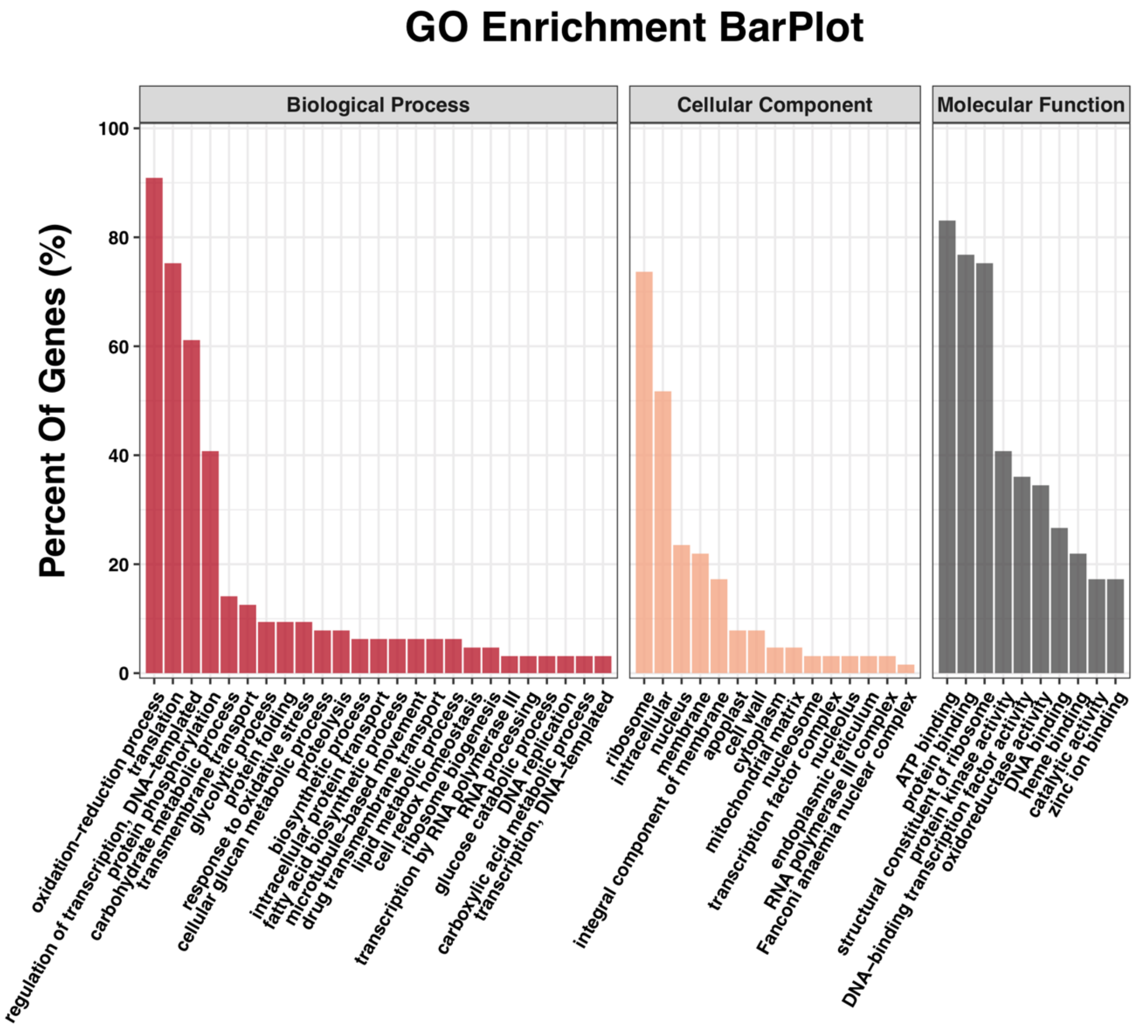

S3-E: 0 profile-white GO Term

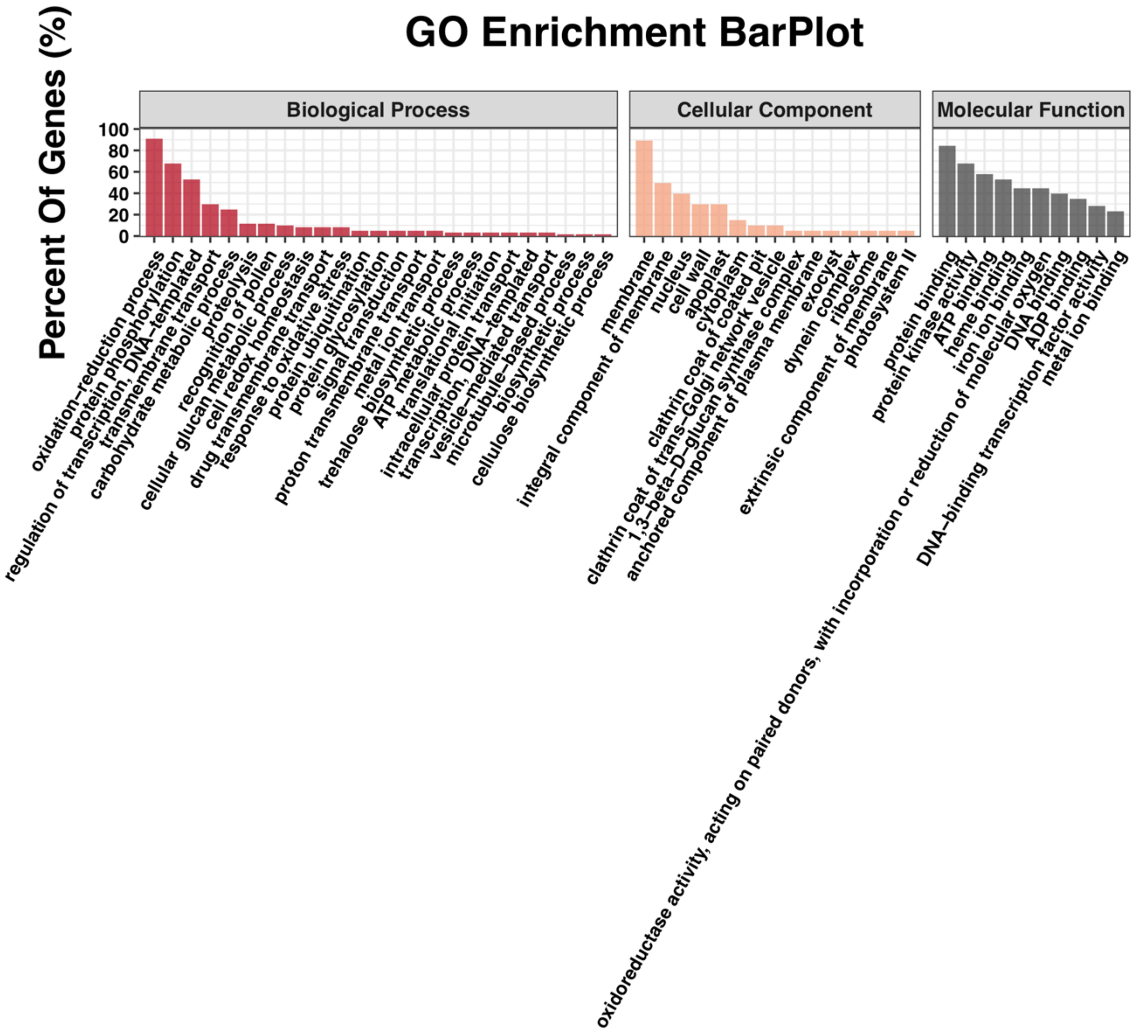

S3-F: 19 profile-white GO Term

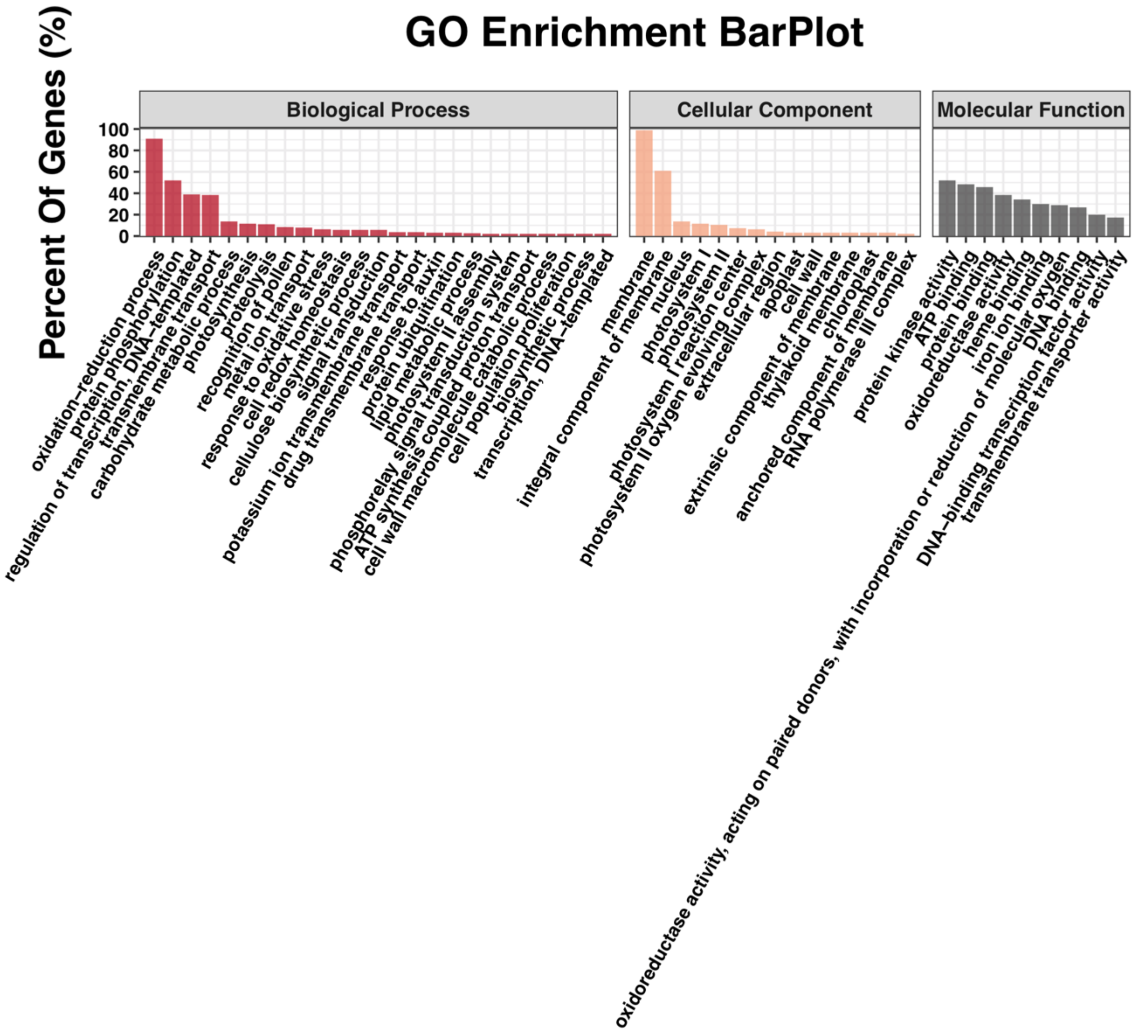

S3-G: 17 profile-white GO Term

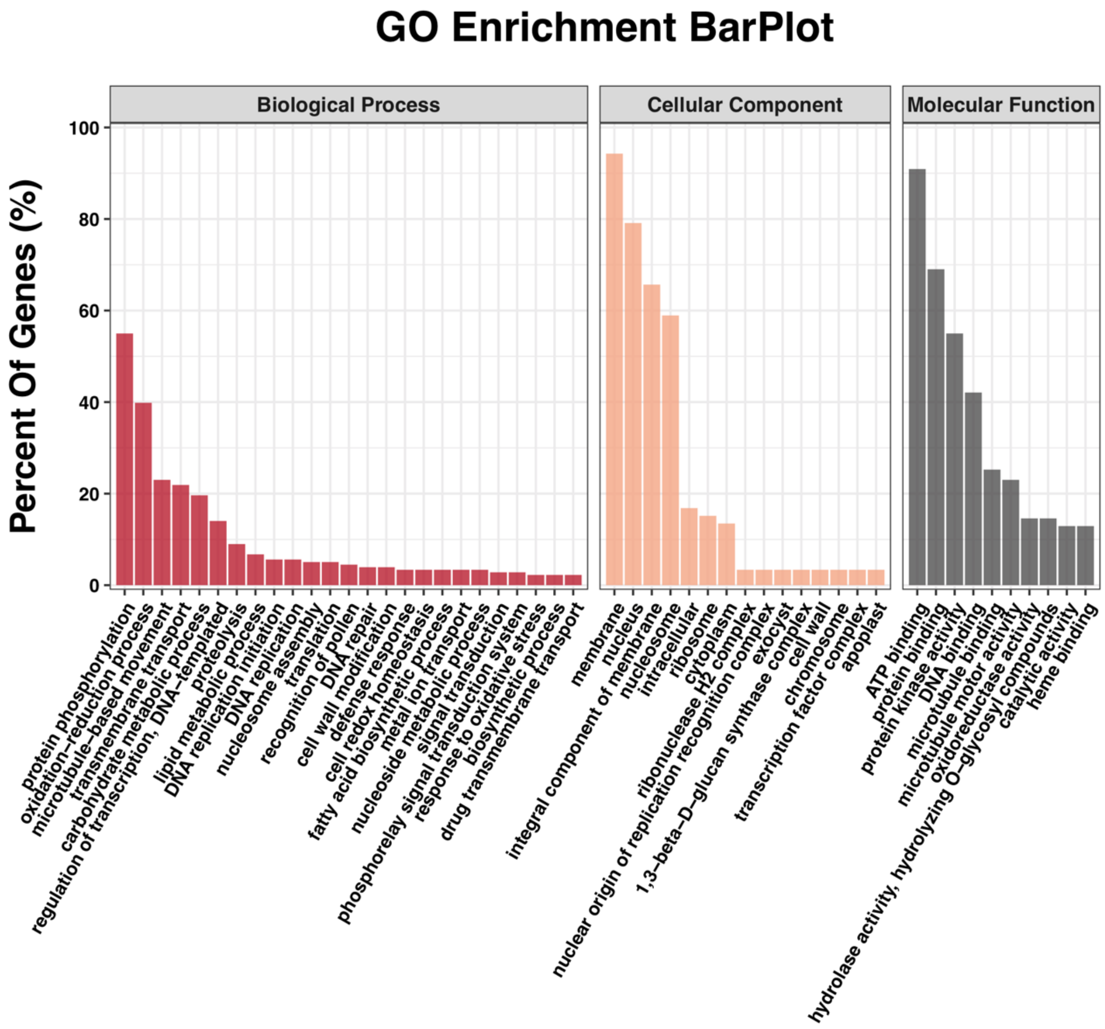

S3-H: 4 profile-white GO Term

FIGURE S3 GO enrichment barplot of 0,19,17,4 profiles under RL and WL .(A) GO enrichment barplot of 0 profile under RL. (B) GO enrichment barplot of 19 profile under RL. (C) GO enrichment barplot of 17 profile under RL. (D) GO enrichment barplot of 4 profile under RL. (E) GO enrichment barplot of 0 profile under WL. (F) GO enrichment barplot of 19 profile under WL. (G) GO enrichment barplot of 17 profile under WL. (H) GO enrichment barplot of 4 profile under WL.

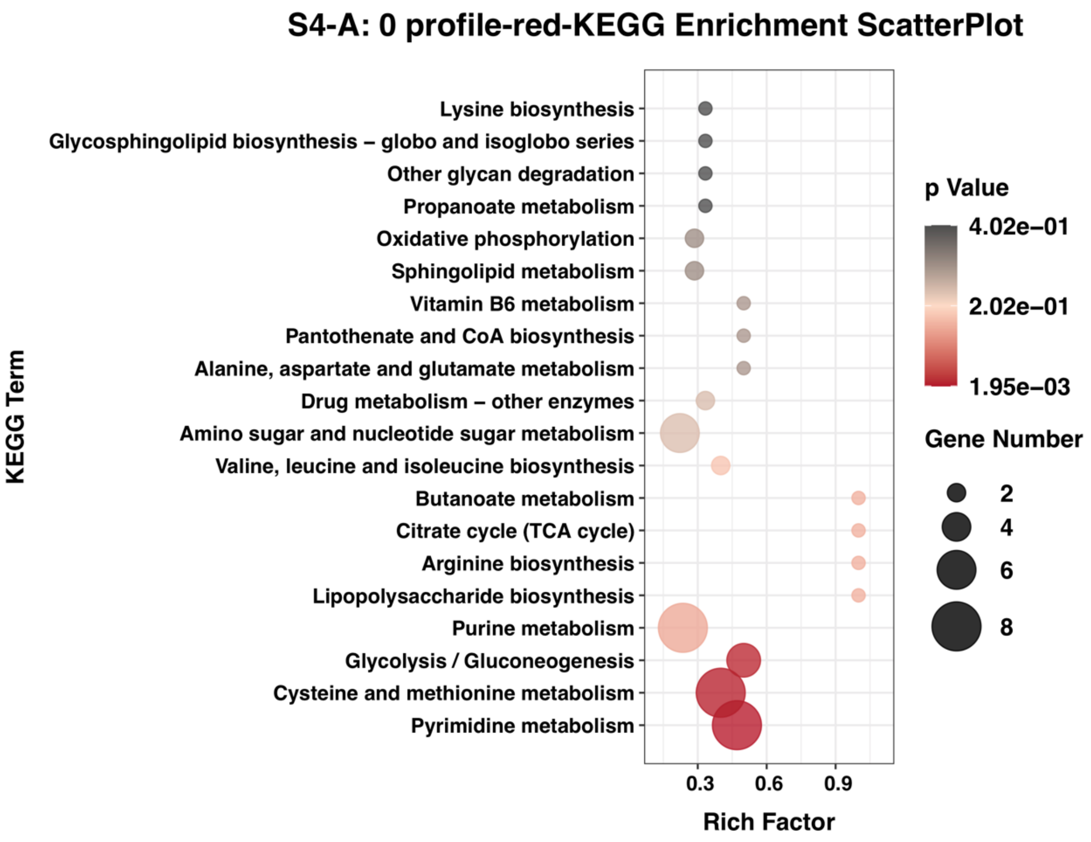
S4-A: 0 profile-red-KEGG Enrichment ScatterPlot
KEGG Term
Lysine biosynthesis
Glycosphingolipid biosynthesis – globo and isoglobo series
Other glycan degradation
Propanoate metabolism
Oxidative phosphorylation
Sphingolipid metabolism
Vitamin B6 metabolism
Pantothenate and CoA biosynthesis
Alanine, aspartate and glutamate metabolism
Drug metabolism – other enzymes
Amino sugar and nucleotide sugar metabolism
Valine, leucine and isoleucine biosynthesis
Butanoate metabolism
Citrate cycle (TCA cycle)
Arginine biosynthesis
Lipopolysaccharide biosynthesis
Purine metabolism
Glycolysis / Gluconeogenesis
Cysteine and methionine metabolism
Pyrimidine metabolism
0.3
0.6
0.9
Rich Factor
p Value
4.02e–01
2.02e–01
1.95e–03
Gene Number
2
4
6
8

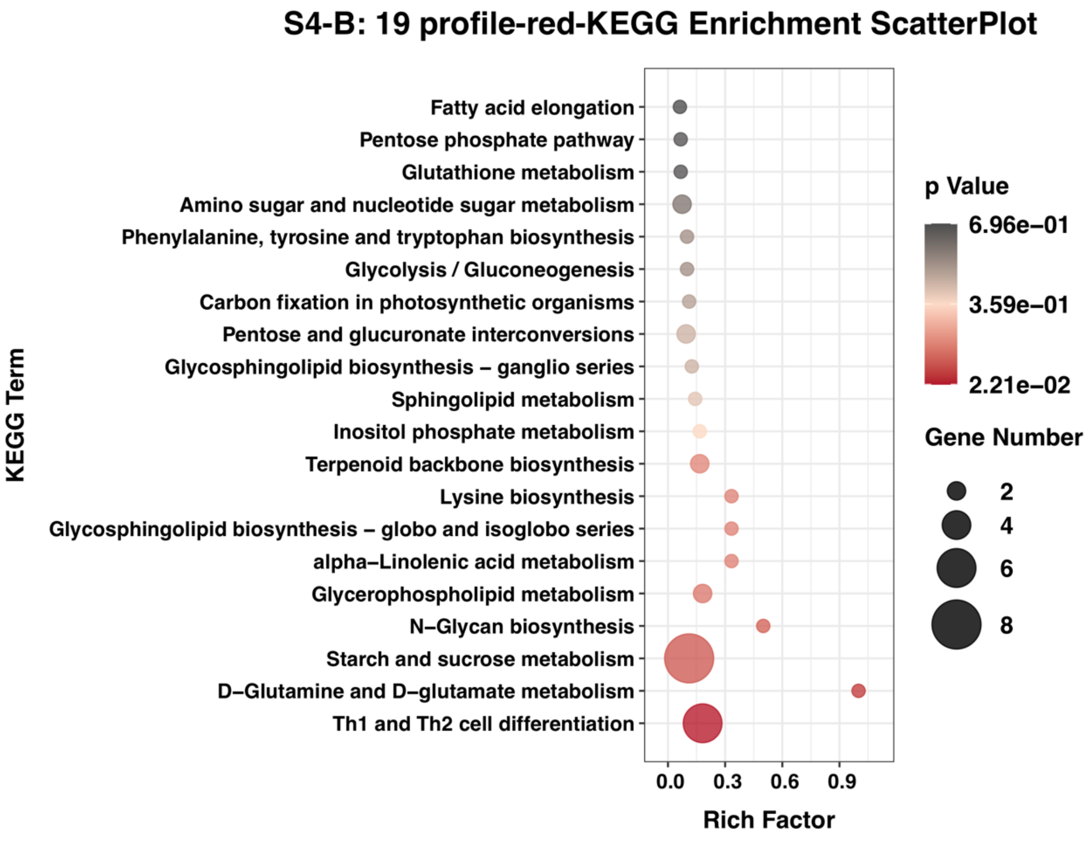
S4-B: 19 profile-red-KEGG Enrichment ScatterPlot
KEGG Term
Fatty acid elongation
Pentose phosphate pathway
Glutathione metabolism
Amino sugar and nucleotide sugar metabolism
Phenylalanine, tyrosine and tryptophan biosynthesis
Glycolysis / Gluconeogenesis
Carbon fixation in photosynthetic organisms
Pentose and glucuronate interconversions
Glycosphingolipid biosynthesis – ganglio series
Sphingolipid metabolism
Inositol phosphate metabolism
Terpenoid backbone biosynthesis
Lysine biosynthesis
Glycosphingolipid biosynthesis – globo and isoglobo series
alpha–Linolenic acid metabolism
Glycerophospholipid metabolism
N–Glycan biosynthesis
Starch and sucrose metabolism
D–Glutamine and D–glutamate metabolism
Th1 and Th2 cell differentiation
0.0
0.3
0.6
0.9
Rich Factor
p Value
6.96e–01
3.59e–01
2.21e–02
Gene Number
2
4
6
8

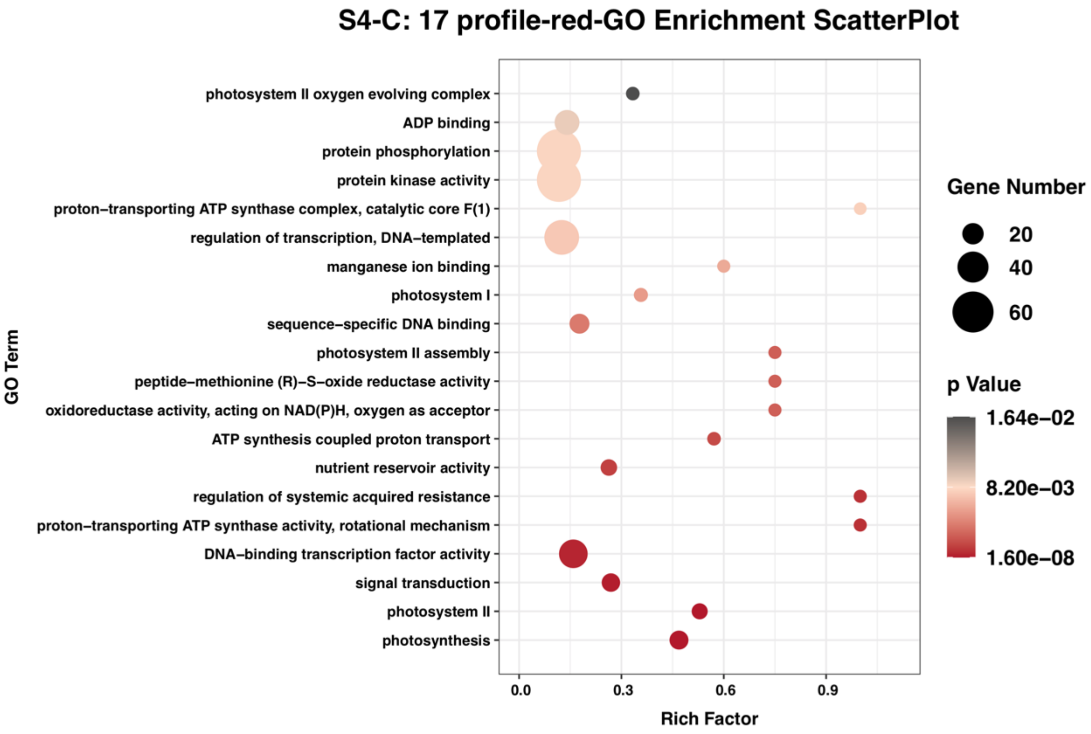

S4-C: 17 profile-red-GO Enrichment ScatterPlot
photosystem II oxygen evolving complex
ADP binding
protein phosphorylation
protein kinase activity
proton−transporting ATP synthase complex, catalytic core F(1)
regulation of transcription, DNA−templated
manganese ion binding
photosystem I
sequence−specific DNA binding
photosystem II assembly
peptide−methionine (R)−S−oxide reductase activity
oxidoreductase activity, acting on NAD(P)H, oxygen as acceptor
ATP synthesis coupled proton transport
nutrient reservoir activity
regulation of systemic acquired resistance
proton−transporting ATP synthase activity, rotational mechanism
DNA−binding transcription factor activity
signal transduction
photosystem II
photosynthesis
GO Term
0.0
0.3
0.6
0.9
Rich Factor
Gene Number
20
40
60
p Value
1.64e−02
8.20e−03
1.60e−08

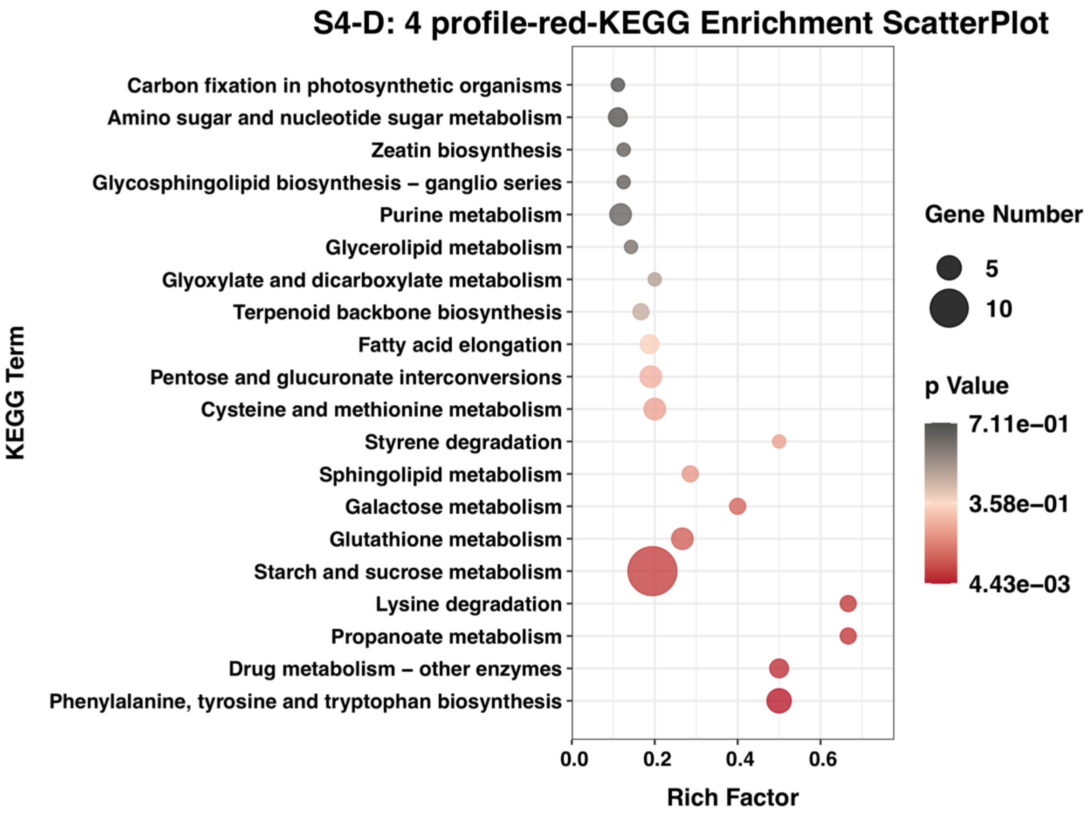

S4-D: 4 profile-red-KEGG Enrichment ScatterPlot
Carbon fixation in photosynthetic organisms
Amino sugar and nucleotide sugar metabolism
Zeatin biosynthesis
Glycosphingolipid biosynthesis − ganglio series
Purine metabolism
Glycerolipid metabolism
Glyoxylate and dicarboxylate metabolism
Terpenoid backbone biosynthesis
Fatty acid elongation
Pentose and glucuronate interconversions
Cysteine and methionine metabolism
Styrene degradation
Sphingolipid metabolism
Galactose metabolism
Glutathione metabolism
Starch and sucrose metabolism
Lysine degradation
Propanoate metabolism
Drug metabolism − other enzymes
Phenylalanine, tyrosine and tryptophan biosynthesis
KEGG Term
0.0
0.2
0.4
0.6
Rich Factor
Gene Number
5
10
p Value
7.11e−01
3.58e−01
4.43e−03

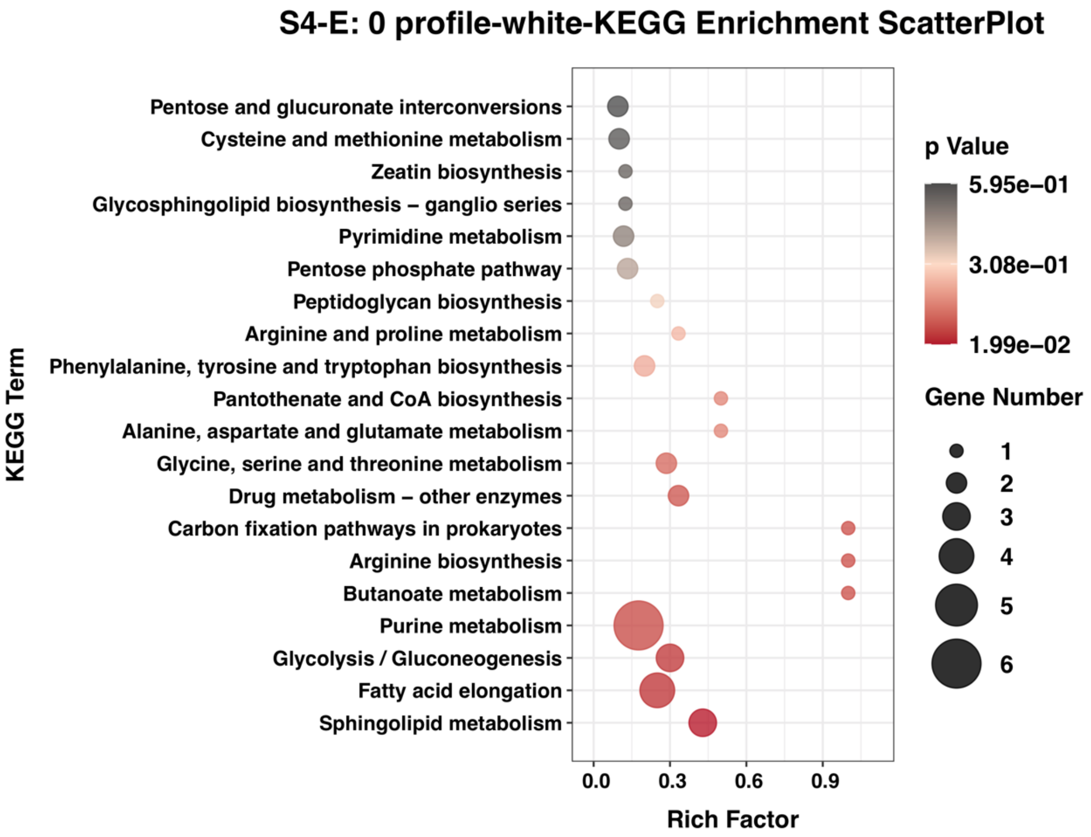
S4-E: 0 profile-white-KEGG Enrichment ScatterPlot
KEGG Term
Pentose and glucuronate interconversions
Cysteine and methionine metabolism
Zeatin biosynthesis
Glycosphingolipid biosynthesis – ganglio series
Pyrimidine metabolism
Pentose phosphate pathway
Peptidoglycan biosynthesis
Arginine and proline metabolism
Phenylalanine, tyrosine and tryptophan biosynthesis
Pantothenate and CoA biosynthesis
Alanine, aspartate and glutamate metabolism
Glycine, serine and threonine metabolism
Drug metabolism – other enzymes
Carbon fixation pathways in prokaryotes
Arginine biosynthesis
Butanoate metabolism
Purine metabolism
Glycolysis / Gluconeogenesis
Fatty acid elongation
Sphingolipid metabolism
0.0
0.3
0.6
0.9
Rich Factor
p Value
5.95e−01
3.08e−01
1.99e−02
Gene Number
1
2
3
4
5
6

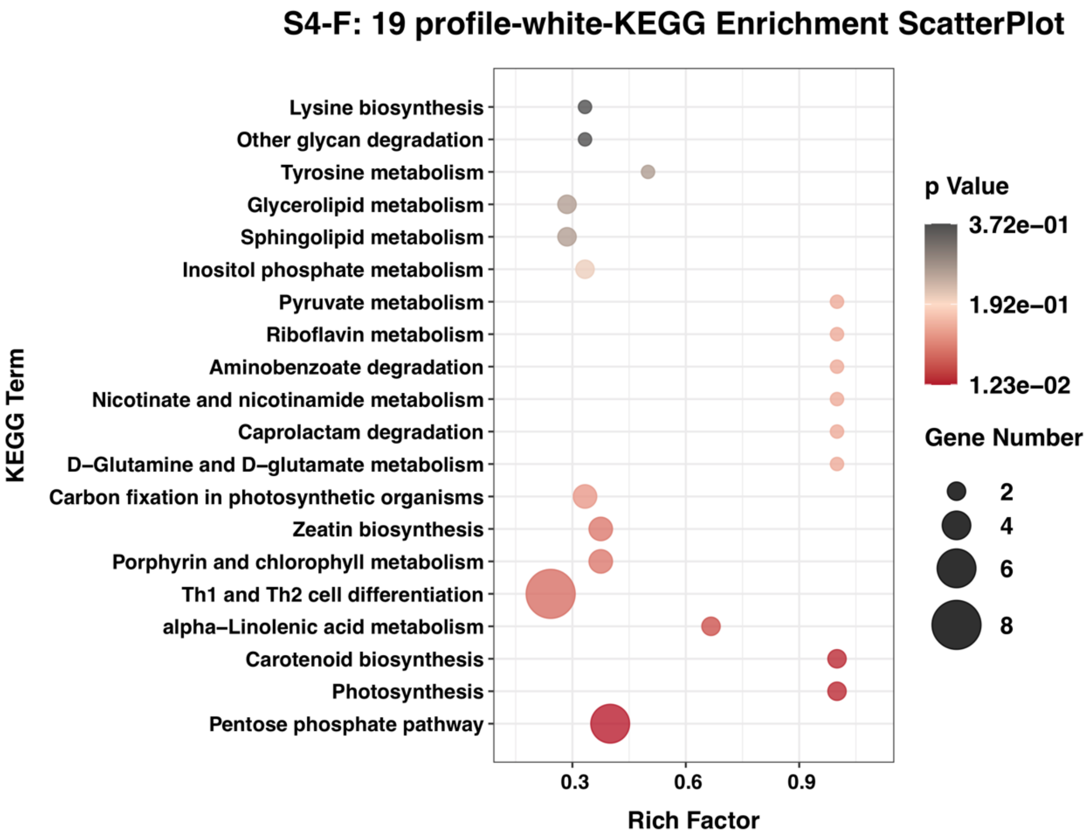
S4-F: 19 profile-white-KEGG Enrichment ScatterPlot
KEGG Term
Lysine biosynthesis
Other glycan degradation
Tyrosine metabolism
Glycerolipid metabolism
Sphingolipid metabolism
Inositol phosphate metabolism
Pyruvate metabolism
Riboflavin metabolism
Aminobenzoate degradation
Nicotinate and nicotinamide metabolism
Caprolactam degradation
D−Glutamine and D−glutamate metabolism
Carbon fixation in photosynthetic organisms
Zeatin biosynthesis
Porphyrin and chlorophyll metabolism
Th1 and Th2 cell differentiation
alpha−Linolenic acid metabolism
Carotenoid biosynthesis
Photosynthesis
Pentose phosphate pathway
0.3
0.6
0.9
Rich Factor
p Value
3.72e−01
1.92e−01
1.23e−02
Gene Number
2
4
6
8

S4-G: 17 profile-white-KEGG Enrichment ScatterPlot

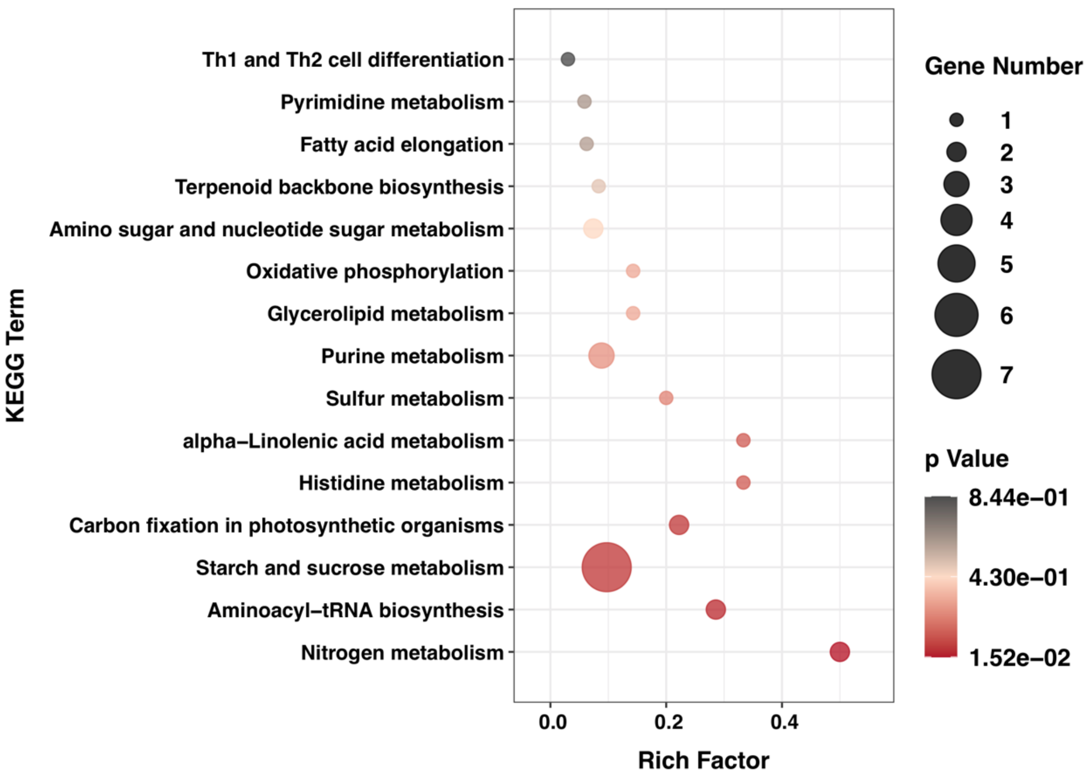

S4-H: 4 profile-white-KEGG Enrichment ScatterPlot

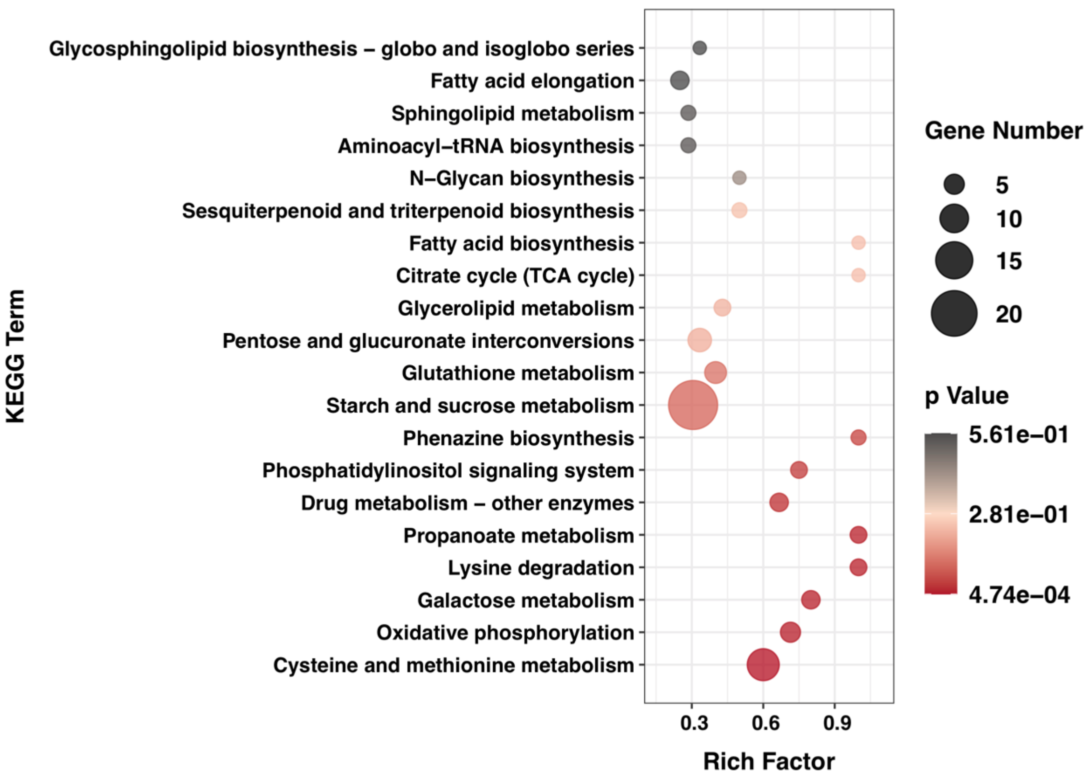

FIGURE S4 KEGG enrichment scatterplot of 0,19,17,4 profiles under RL and WL .(A) KEGG enrichment scatterplot of 0 profile under RL. (B) KEGG enrichment scatterplot of 19 profile under RL. (C) KEGG enrichment scatterplot of 17 profile under RL. (D) KEGG enrichment scatterplot of 4 profile under RL. (E) KEGG enrichment scatterplot of 0 profile under WL. (F) KEGG enrichment scatterplot 19 profile under WL. (G) KEGG enrichment scatterplot of 17 profile under WL. (H) KEGG enrichment scatterplot of 4 profile under WL.

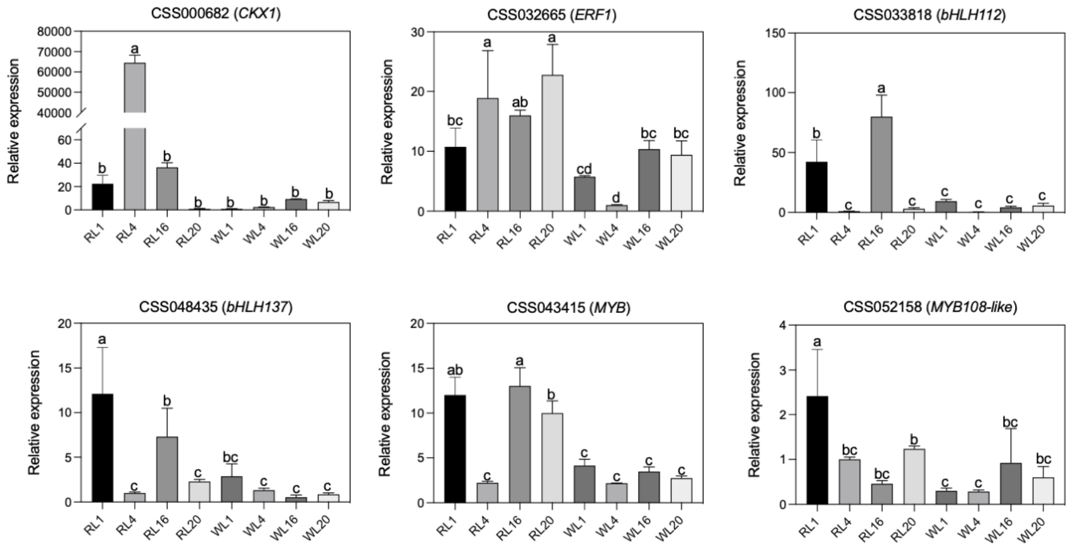

FIGURE S5 Differential expression of CKX1, ERF1, bHLH112, MYB and MYB108-like under red and white light during AR formation and development.
